# Double diffusion for the programmable spatiotemporal patterning of multi-domain supramolecular gels†

**DOI:** 10.1039/d1sc03155d

**Published:** 2021-08-18

**Authors:** Hannah S. Cooke, Lisa Schlichter, Carmen C. Piras, David K. Smith

**Affiliations:** Department of Chemistry, University of York Heslington York YO10 5DD UK David.smith@york.ac.uk

## Abstract

To achieve spatial resolution of a multi-component gel, a double diffusion approach is used which enables the precise programming of self-assembled patterned domains with well-defined shapes and sizes. The low-molecular-weight gelators (LMWGs) used in this study are pH-responsive DBS-CO_2_H and thermally-responsive DBS-CONHNH_2_ (both based on 1,3:2,4-dibenzylidenesorbitol, DBS). A DBS-CONHNH_2_ gel was initially assembled in a tray, and then loaded at carefully-selected positions with either basified DBS-CO_2_H (*i.e.* DBS-carboxylate) or an acid. These soluble components subsequently diffuse through the pre-formed gel matrix, and in the domains when/where they mix, protonation of the DBS-carboxylate induces self-assembly of the DBS-CO_2_H network, leading to a patterned gel-in-gel object with well-defined shape and dimensions. Using a strong acid achieves fast gelation kinetics, creating smaller, better-defined macroscale objects but with less nanoscale order. Using a weak acid source with slow kinetics, gives slightly larger objects, but on the nanoscale the DBS-CO_2_H network formation is better controlled, giving more homogeneous nanoscale structures and stiffer objects. The patterned objects can be further reinforced by the presence of agarose polymer gelator. The shape of the patterning is programmed by both the shape of the central reservoir and the starting geometry in which the reservoirs are organised, with the balance between factors depending on assembly kinetics, as dictated by the choice of acid. This simple methodology therefore enables programming of patterned gels with spatiotemporal control and emergent patterning characteristics.

## Abstract 2

To achieve spatial resolution of a multi-component gel, a double diffusion approach is used which enables the precise programming of emergent self-assembled patterned domains with well-defined shapes and sizes.

## Graphical abstract

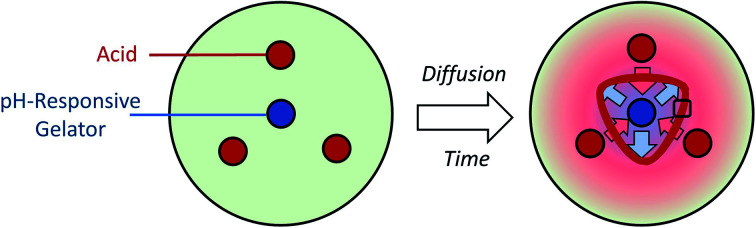

## Introduction

Supramolecular gels are formed when low-molecular-weight gelator (LMWG) building blocks spontaneously assemble into fibrillar networks as a result of non-covalent interactions.^1^ The dynamic and responsive nature of these gels makes them of use in a range of different technologies.^2^ In general, supramolecular gels are relatively weak and unstructured, but recently, efforts have been made to shape, structure or pattern such gels,^3^ using techniques including 3D-printing^4^ and photopatterning.^5^

One approach to patterning gels uses controlled diffusion. In a key study, van Esch, Eelkema and co-workers diffused aldehyde and acylhydrazide components from separate reservoirs within a pre-formed polymer gel matrix. This gave rise to self-standing gel objects in the locations where the two components met and reacted to form a self-assembling LMWG, with the loading geometry controlling the shape.^6^ In other work, our research group used the diffusion of two components across a gel–gel interface, that then combined to form a gel to yield interpenetrated gels.^7^

There has also been interest in diffusing acids to trigger LMWG assembly. In early work, a diffusing acid was used to assemble an LMWG, with gel fibrils aligning with the propagating acidic diffusion wave.^8^ Besenius, Hermans and co-workers used polymer cubes pre-soaked in HCl and showed that a pH-responsive LMWG on the surface underwent controlled gel assembly, with the chemical composition of the polymer cube controlling the kinetics of acid diffusion out of the gel cube and hence gel assembly.^9^ Spatial patterning of an acidic catalyst on a surface has been used to achieve spatially directed LMWG assembly,^10^ as has localised electrochemical production of H^+^ on an electrode surface.^11^ Microfluidics can also bring an acid trigger into spatially-controlled contact with an LMWG.^12^

There has recently been increasing interest in kinetic and dynamic aspects of gel assembly. For example, van Esch and co-workers used an alkylating agent as fuel to generate an ester LMWG, while the process was reversed by ambient hydrolysis.^13^ A number of reports have shown that hydrogels can exist in non-equilibrium states, with temporal control increasingly being combined with a degree of spatial resolution.^14^ In this regard, a photo-acid has been used to create a gradient of acid, leading to a gel with a transient gradient of LMWG network assembly, and hence stiffness, that evolves over time.^15^

There is considerable interest in using multiple components to assemble gels in order to incorporate additional function.^16^ For some time, we have been using multi-component gels to achieve patterned gel-in-gel assembly. For example, using a photoacid enabled us to achieve photo-patterned assembly of a pH-responsive gelator (carboxylic acid-modified 1,3:2,4-dibenzylidenesorbitol, DBS-COOH) in other pre-formed self-assembled gels, *e.g.* thermally-responsive DBS-CONHNH_2_ gel.^17^

**Fig. 1:**
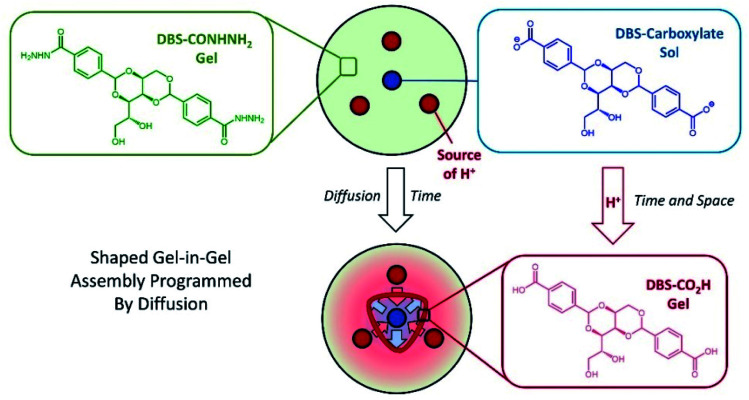
Schematic of diffusion experiment in which DBS-carboxylate diffuses out from one reservoir cut into a pre-assembled DBS-CONHNH_2_ gel, and meets an acid diffusing from other reservoir(s). This leads to dynamic protonation to give a self-assembled DBS-CO_2_H network with spatial control. The end result is programmed by the shape of the central reservoir, the starting geometry of the outer reservoirs, and aspects such as acid choice and concentration.

We built on this work and recently demonstrated that diffusing an acid trigger through the pre-formed DBS-CONHNH_2_ gel matrix could be an effective way of directing assembly of the DBS-COOH secondary gel network, creating materials with spatial resolution that can also temporally evolve.^18^ Having established that diffusion of an acid from a central reservoir could direct gel-in-gel assembly with radial spatial control, we turned our attention to assembling patterned gel-in-gel objects with better-defined shapes. Inspired by van Esch and co-workers,^6^ we reasoned that incorporating two diffusing soluble components into gels may be a way of achieving this. However, unlike their use of two tailored reagents to form the gelator, one of our diffusing species is just a simple acid, with the other being the deprotonated LMWG – gelation occurs when the two species meet, simply as a result of protonation (Fig. 1). Given the prevalence of acid-triggered gels in the literature, our approach may be of general applicability. This paper reports our investigations using this approach, in which we develop ground rules and demonstrate how this process can be kinetically tuned and controlled to create patterned gel objects with controllable rheological properties and a high degree of spatial resolution. Precisely-shaped gels assembled from LMWGs remain very rare, but in the longer term have potential applications in areas such as tissue engineering.^19^

## Results and discussion

### Patterning of gel trays by double diffusion

**Fig. 2:**
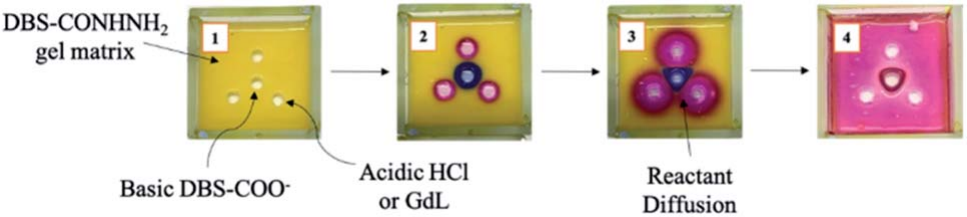
Fabrication of a patterned gel by diffusion control through a pre-formed DBS-CONHNH_2_ matrix containing thymol blue (TB) indicator. (1) Addition of the gelator precursors, DBS-carboxylate and HCl or GdL; (2) independent diffusion of the precursors, DBS-carboxylate (blue), HCl (pink), GdL (not shown, yellow); (3) crossing of the diffusional fronts after a period of time; (4) system after equilibration and full diffusion, showing the multi-component object that assembled at the points where the diffusional fronts crossed (red).

The two LMWGs were synthesised using our well-established methods.^20^ A DBS-CONHNH_2_ gel containing thymol blue (TB) pH indicator was then prepared in a 5 mL tray (5 × 5 cm), spatially defined holes/reservoirs were cut into the gel and loaded with the two DBS-CO_2_H gelator precursors (Fig. 2 and S1†). In our first experiment, we loaded the central reservoir with basified DBS-carboxylate, while we loaded three outer reservoirs with an acid – either HCl or glucono-δ-lactone (GdL). We selected HCl as an archetypal strong acid with fast kinetics, and chose GdL because it hydrolyses with slower kinetics to give a weak acid, in a process that is known to give rise to controlled and very effective assembly of acid-functionalised LMWGs into highly homogeneous gels.^21^

After loading, the reactants subsequently diffused through the pre-formed DBS-CONHNH_2_ matrix. The basic DBS-carboxylate solution was indicated blue by the TB. GdL was indicated yellow (the same colour as the DBS-CONHNH_2_ gel itself), and HCl pink. Upon crossing of the diffusional fronts, a pH change was indicated by TB through a blue to yellow (pH 9.6–8.0) colour change, which in the case of HCl, further progressed from yellow to pink (pH 2.8–1.2). This indicated the acidification of DBS-carboxylate, with resulting formation and self-assembly of DBS-CO_2_H to give a slightly opaque hybrid gel. When using three outer reservoirs, the self-assembled domain of DBS-CO_2_H was a curved triangle delineating the interface at which the two diffusion fronts meet one another. The positions of the starting reservoirs therefore appear to program the resulting object in a similar way as previously described by van Esch and co-workers for the formation of acylhydrazone-based gels by diffusion.^6^

#### Effect of acid concentration

The identity and concentration of the acid precursor were hypothesised to affect the rate and efficiency of DBS-carboxylate acidification, and it was reasoned that this might impact on gel patterning. To assess this, HCl and GdL were each employed at a range of different loading concentrations. In this paper, we present acid loadings, rather than solution concentrations – *i.e.*, we state the number of mmoles of acid loaded into the reservoirs (units: mmol). The reservoirs are very small, so these loadings are not the actual solution ‘concentrations’, which are significantly higher, but they are the most relevant values to discuss.

**Fig. 3:**
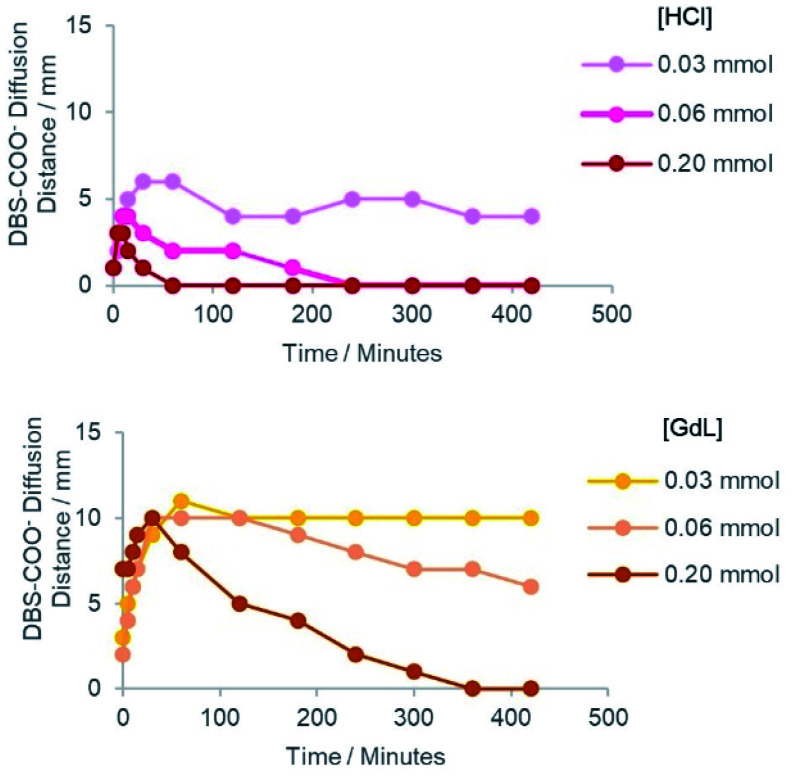
The distance of DBS-carboxylate diffusion from the central reservoir, as indicated by the blue colour, over a period of 7 hours (420 min). Graphs shown are (top) HCl and (bottom) GdL at different loadings (see legend). For these data, the reservoirs were cut in a square pattern to give a symmetric radial diffusion pattern.

In each case, the DBS-carboxylate initially diffused out from the central reservoir, prior to its acidification and assembly – this could be visualised by an expanding domain of yellow to blue colour change. The distance of diffusion over time is plotted in Fig. 3. As the diffusional fronts progressed and the acid came into contact with the DBS-carboxylate, the two precursors reacted and the gel pH then locally decreased again, indicated by a blue to yellow colour change – the basic region then began to shrink in size. This reflects the fact that as DBS-carboxylate is protonated, its diffusion stops as the resulting DBS-CO_2_H gets immobilised into the solid-like self-assembled network. In general, at higher loadings of acid, the DBS-carboxylate did not diffuse as far into the supporting gel before being protonated and assembled – in such cases the acid was also better able to fully convert the pH indicator from blue back to yellow or red (Fig. 3).

When HCl was used as the acid, the neutralisation of DBS-carboxylate by acidification was rapidly achieved, with the DBS-carboxylate diffusional front reaching its maximum diameter in just *ca.* 10–30 minutes, with maximum diameters of *ca.* 3–6 mm being achieved, depending on HCl loading. As expected, higher acid loadings more effectively limited the diffusion of DBS-carboxylate.

In contrast, when GdL was loaded into the outer reservoirs, not only does the GdL need to diffuse, but it also needs to hydrolyse to release gluconic acid – this slows down the acidification kinetics. In this case, the DBS-carboxylate is typically able to diffuse to *ca.* 10 mm diameter in each case. The rate of subsequent acidification then increased with increasing GdL loading.

For HCl, the fact it is a strong acid ensures the kinetics of gelation are faster than the kinetics of mixing, resulting in immediate acidification and DBS-CO_2_H assembly. With GdL, the kinetics of mixing are faster than of hydrolysis and gelation – significant mixing of the two precursor solutions occurred before any pH reduction. Thus, there was a time delay in DBS-carboxylate acidification to *ca.* 30–60 minutes. In this way, the patterning process can be kinetically tuned. This has an impact on the sizes and shapes of the patterned objects that can be generated with the two different acids (see below).

#### Effect of a polymer gel additive

We also formed gels in the presence of agarose (1% wt/vol), a polymer gelator (PG) which acts as a robust thermally-induced supporting matrix. Agarose can be present in such systems without adversely affecting the self-assembly of DBS-CONHNH_2_ or DBS-CO_2_H.^20*a*,22^

The influence of agarose on DBS-carboxylate diffusion was probed using the same approaches as described above. Overall, the diffusion processes were broadly similar in the presence of agarose (see Fig. S4 and S5†). However, it did appear that when agarose was incorporated, the initial DBS-carboxylate diffusion distance was slightly smaller, suggesting that the presence of the agarose PG may have some limited impact on the diffusion of this species, either as a result of greater network density, or a degree of interaction with the diffusing species.

#### Effect of loading geometry

**Fig. 4:**
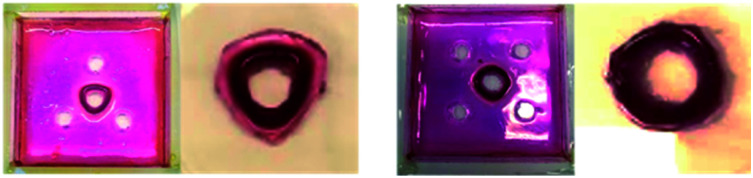
The formation of patterned gels based on initial loading geometries with HCl reservoirs patterned around a central DBS-carboxylate reservoir, that are either: (left) triangular or (right) square. The hybrid gel regions have been manually cut out of the multi-component gel trays at the end of the experiment to emphasise their geometries, which are (left) curved triangular and (right) roughly circular.

We then compared the triangular pattern of external reservoirs discussed above with a square pattern (Fig. S2 and S6–S9†). In this case, rather than forming a curved triangular patterned hybrid gel object, in which the influence of the three external reservoirs could clearly be seen, when using HCl we instead fabricated a roughly circular hybrid gel object (Fig. 4 and S3†). In this way, the initial loading geometry clearly programs the outcomes in terms of spatial patterning. We had initially considered that a curved square was the likely outcome from this pattern of reservoirs, however, we hypothesise that once there are this number of external reservoirs, the shape of the object formed primarily corresponds to the shape of the central reservoir, which is circular in nature and is less influenced by the precise locations of the external acid reservoirs – this indicates that with four external reservoirs, the acid essentially approaches the central reservoir from ‘all’ directions roughly equally. However, when there are only three external reservoirs, the directionality of those reservoirs relative to the central reservoir matters, as they represent the main directions of flux of the diffusing acid, and as such, the external reservoirs impose more of their geometric programming onto the resulting patterns. Studies described below using a range of different patterns and different acids confirm this hypothesis, and also demonstrate that the patterning process can be kinetically controlled.

**Fig. 5:**
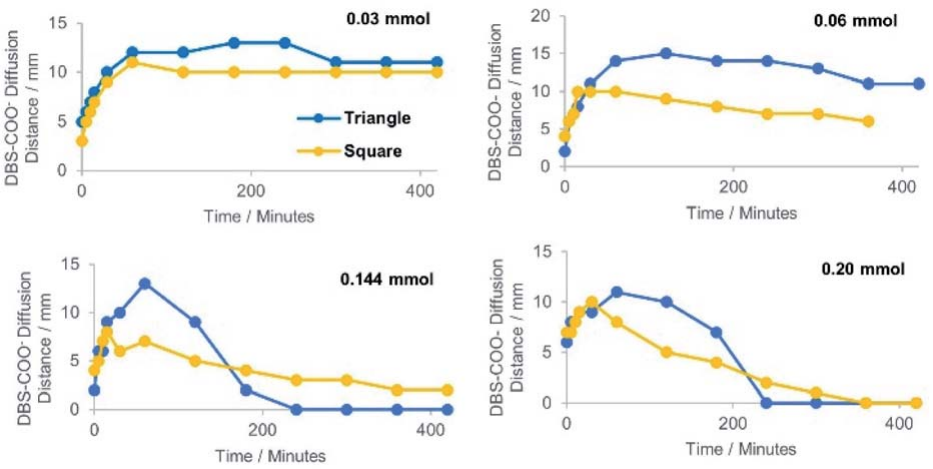
The distance of DBS-carboxylate diffusion from the central reservoir, as indicated by a colour change, over a period of 7 hours (420 min) at different loadings of HCl using either a triangular or square geometry.

To further understand the temporal differences between the triangular and circular loading patterns, the rates and extents of diffusion and acidification were determined using different loadings of HCl (Fig. 5). In general, the acidification was more efficient with the square pattern, represented by a shorter diffusion distance of DBS-carboxylate at each acid loading and a more rapid neutralisation. This reflects a greater capacity for acidification with an increasing number of external acid-loaded reservoirs. As expected, the differences between loading geometries were smallest at low acid loadings and became more pronounced as the acid loading increased.

**Fig. 6:**
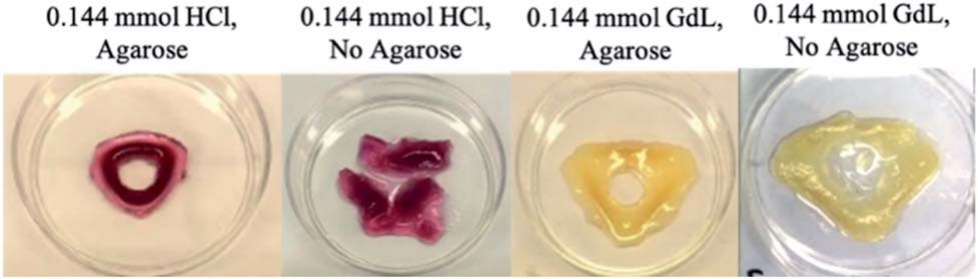
Free-standing objects fabricated from the 0.144 mmol HCl_(triangle)_ hybrid and LMWG gels, and 0.144 mmol GdL_(triangle)_ hybrid and LMWG gels. The softer LMWG systems, particularly that formed using HCl, segmented at the corners where mechanical strength was weaker.

Attempts were made to cut the resulting gel objects out of the supporting medium. However, in general, the patterned gels were soft and structurally fragile and this led to breakage – for example, the triangular patterns described above tended to fragment at their corners (Fig. 6). However, in the presence of agarose PG, once diffusion and assembly was complete, the structures generated could be cut out of the surrounding gel matrix to facilitate visual study. Each object contained a central hole, and appeared darker and stiffer than the surrounding gel, indicating the spatially-defined formation of the DBS-CO_2_H network (see below for more detailed characterisation). The colour of the object was defined by the response of the indicator to gel pH, with HCl, the stronger acid, fabricating pink objects and GdL giving yellow structures.

As described above, as the acid loading increased, the DBS-carboxylate diffused less far from the central reservoir before being acidified. This impacts on the patterned objects, which became smaller, forming closer to the central hole. On the other hand, for GdL, its slow hydrolysis facilitated extended DBS-carboxylate diffusion, resulting in a somewhat larger structure, further from the central hole. In summary, when using a stronger acid with faster gelation kinetics, smaller objects with greater spatial resolution were fabricated.

**Fig. 7:**
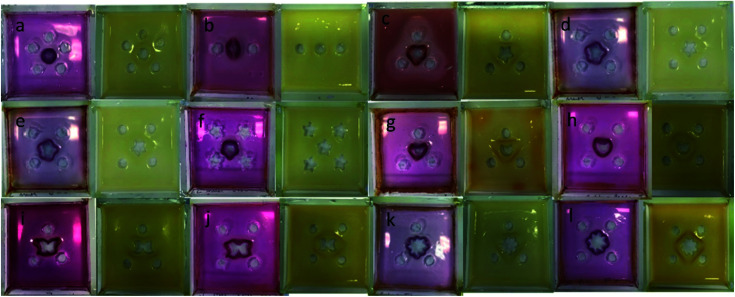
More complex hydrogel patterns fabricated using the 0.20 mmol HCl (pink) and GdL (yellow) hybrid gels with DBS-carboxylate loaded into the central reservoir. Gel objects (not pictured) could be obtained by cutting and removing the structures from the surrounding gel. (a) Circle surrounded by 5 circles, (b) circle surrounded by 2 circles, (c) star surrounded by 3 circles, (d) star surrounded by 4 circles, (e) star surrounded by 4 circles, (f) star surrounded by 4 stars, (g) moon surrounded by 3 circles, (h) moon surrounded by 4 circles, (i) butterfly surrounded by 3 circles, (j) butterfly surrounded by 4 circles, (k) flower surrounded by 3 circles, (l) flower surrounded by 4 circles.

More complex shapes were easily fabricated using this hybrid gel approach simply by varying the loading geometry. Fig. 7 illustrates a selection of examples of the architectural intricacy achieved, and clearly demonstrates the feasibility of manipulating and shaping hydrogels in this way. The use of two outer acid reservoirs on either side of the central DBS-carboxylate reservoir led to a pattern with two slightly separated hemispherical arcs of DBS-CO_2_H as the acid diffuses towards the DBS-carboxylate from left and right sides of the tray, but not from the top and bottom. Cutting differently-shaped central reservoirs within the triangular pattern led to modified output patterns. When HCl was present in the external reservoirs (pink patterns), the star and moon maintained approximately triangular-patterned DBS-CO_2_H assemblies, albeit slightly modified by the shape of the central reservoir. However, as the central reservoir became more intricate (*e.g.* flowers and butterflies), it increasingly directed the pattern of the resulting DBS-CO_2_H assembly. This indicates that having three external reservoirs is borderline between diffusion of the HCl from the external reservoirs dictating the pattern, and diffusion of DBS-carboxylate from the central reservoir taking control. Interestingly, however, with GdL in the external reservoirs (yellow patterns), all the triangular patterns gave rise to broadly triangular objects irrespective of the shape of the central reservoir. This reflects the fact that GdL diffusion from the outer reservoirs and slow hydrolysis become the controlling feature in the assembly process, and hence the external reservoirs dictate the resulting geometry rather than the shape of the central DBS-carboxylate reservoir.

For square patterns, when HCl is in the four external reservoirs, the shape of the central reservoir always dictated the output. In the same way that, as described above, a circular central hole resulted in a circular pattern, the star-, moon-, flower- and butterfly-shaped central reservoirs each led to patterns that replicated their own shape. This reflects the fact that once there are four external reservoirs, the acid is diffusing roughly equally towards the central reservoir from all directions and so diffusion of DBS-carboxylate out of the central reservoir becomes dominant. However, once again, with GdL in the four external reservoirs, the patterns formed in the square loading geometries mostly retained aspects of a square shape. As for the triangular patterns, this reflects the fact that GdL diffusion from the external reservoirs and slow hydrolysis now becomes the controlling feature, with the DBS-carboxylate having moved further from the central reservoir, which therefore exerts less control on the resulting pattern.

Changing the shape of the external reservoirs from circles to stars did not appear to have a major effect on the patterns formed, which indicates they can largely just be considered as an external source of protons.

Finally, we programmed a pattern with a pentagonal array of five external reservoirs around the central reservoir. In this case, both HCl and GdL gave rise to approximately circular patterns, reflecting the central circular DBS-carboxylate loading reservoir. With HCl in the external reservoirs, the pattern was, once again, much more tightly-defined and closer to the central reservoir than with GdL.

In summary therefore, both the shape of the central reservoir and the geometry of the surrounding external reservoirs can control the patterning of the self-assembled DBS-CO_2_H network. When using a strong acid that achieves rapid gel assembly, or when using lots of external reservoirs, the shape of the central reservoir became dominant. However, when using GdL which only slowly releases H^+^, or when using a limited number of external reservoirs, the geometry of the external reservoirs becomes dominant. In this way, the gel shapes obtained are an emergent consequence of the overall experimental conditions rather than a simple direct reflection of the diffusion pattern.

### Characterisation of patterned gels

In the sections above, we largely made use of indicators within the gel to report on diffusion, combined with visual observations of assembled regions. It was vitally important to more fully characterise these patterned gels to understand the process in more detail.

**Fig. 8:**
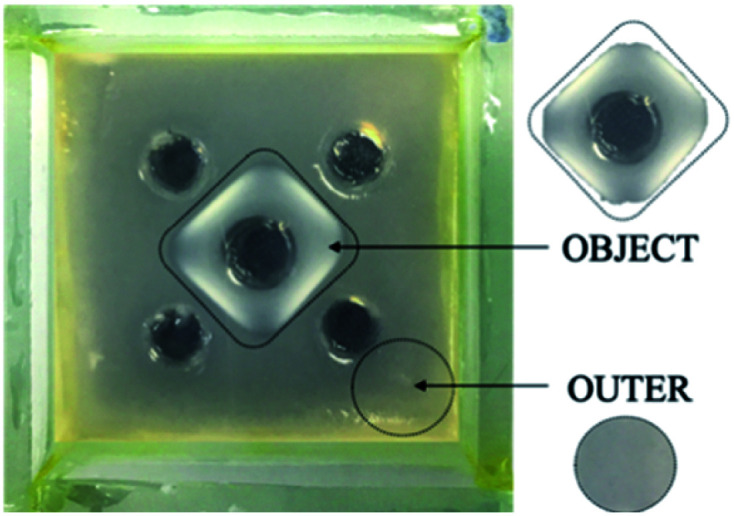
The object and outer gel domain samples obtained for characterisation, shown here for 0.20 mmol GdL_(square)_ hybrid gel in the absence of TB.

For these studies, we made use of square-patterned gels in the absence of indicator – even in such systems the patterned self-assembly zone could easily be visualised (Fig. 8). For the purposes of characterisation, we then defined two different domains of these materials – (i) object and (ii) outer. For characterisation purposes, samples from each of these domains were cut and then removed with a spatula. In principle, the patterned object should contain both self-assembled DBS-CONHNH_2_ and DBS-CO_2_H, while the outer domain should only contain self-assembled DBS-CONHNH_2_. Both domains should have been approximately equivalently exposed to acid diffusing from the external reservoirs.

#### ^1^H NMR spectroscopic characterisation

^1^H NMR spectroscopy was used to determine gelator composition in the hydrogel objects. The objects fabricated with HCl and GdL, at each acid loading using a square loading geometry were studied. The patterned gel objects were prepared, removed from the tray and dried under ambient conditions to form a xerogel. A weighted sample of the xerogel was then dissolved in DMSO-*d*_6_ solvent, and acetonitrile added as an internal standard. By comparing the integrals of the LMWGs in the aromatic region (7–8 ppm) to the integrals of an internal standard (acetonitrile, *δ* = 2.09 ppm), it was possible to calculate the quantity of each LMWG present in the sample (Fig. S10–S14 and Table S1†).

At higher acid loadings, a greater percentage of the DBS-CO_2_H was observed within the object (Table 1). Furthermore, more DBS-CO_2_H was observed in the patterned object when using HCl than when using GdL. At higher loadings of HCl, the diffusion of DBS-carboxylate to the outer domain was limited. This correlated with the smaller, more opaque patterned gel objects, as described above. For GdL (and at low HCl loadings), the percentage of DBS-CO_2_H LMWG observed within the object was lower, implying significant DBS-carboxylate diffusion to the outer domain due to slow or ineffective acidification. In the presence of agarose, broadly similar trends for the incorporation of DBS-CO_2_H into the patterned object were observed (see ESI†).

**Table 1:** Percentage of DBS-CO_2_H LMWG in the xerogel object for HCl and GdL patterned gels

Acid concentration/mmol	HCl	GdL
0.03	21.3%	24.0%
0.06	45.8%	22.6%
0.144	61.4%	45.1%
0.20	75.0%	48.7%

#### IR characterisation

Infrared spectroscopy was used to probe DBS-CO_2_H assembly and the presence of non-covalent interactions within the patterned gel object. The object and outer domain samples of the HCl LMWG gel system, were prepared. The samples were dried to form xerogels, and analysed by IR (Fig. S15–S17 and Table S2†). The C=O stretch is particularly distinctive, and is known to shift from 1640 cm^−1^ for the xerogel of DBS-CONHNH_2_ to 1685 cm^−1^ for the two-component DBS-CONHNH_2_/DBS-CO_2_H gel.^17*b*^ We found a C=O stretch of *ca.* 1650 cm^−1^ in the outer domain and *ca.* 1690 cm^−1^ in the inner domain. Similar changes were observed in the presence of agarose, even though the spectrum was somewhat more complicated by the presence of agarose C=O bands. These results are therefore consistent with the view that DBS-CO_2_H has been self-assembled within the patterned domain. There were also changes in the N–H/O–H region. Although the peaks were broad, the outer domain had a maximum at *ca.* 3150–3200 cm^−1^ while for the patterned gel object, the maximum was at *ca.* 3300–3350 cm^−1^. In the presence of agarose, again the OH bands of agarose somewhat confuse the spectrum, but there was a shift from *ca.* 3150–3300 cm^−1^ for the outer domain to *ca.* 3300–3400 cm^−1^ for the patterned object. Once again, these observations are consistent with the patterned object having a different composition to the outer domain as a result of self-assembly of DBS-CO_2_H within it.

#### TEM and SEM characterisation

Transmission electron microscopy (TEM) and scanning electron microscopy (SEM) were used to study the nanoscale structuring and morphology of the 0.2 mmol HCl and GdL gels. Samples were prepared from both the patterned object and the outer domain. TEM samples were prepared on copper grids then negatively stained with uranyl acetate. SEM samples were freeze-dried at −60 °C in an attempt to minimise the drying effects that can adversely impact on gel morphologies visualised by electron microscopy methods.^23^

**Fig. 9:**
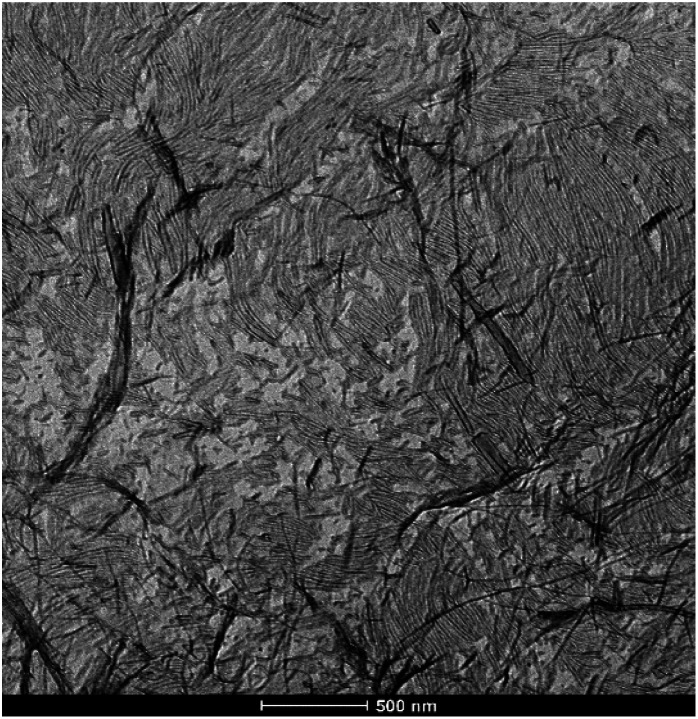
TEM image of the 0.20 mmol GdL hybrid objects showing highly aligned domains of self-assembled nanofibers. Scale bar: 500 nm.

In all cases, a nanofibrillar morphology was observed (Fig. S18 and S19†). In the presence of agarose, the networks that were formed appeared more branched and interpenetrated, as would be expected based on the presence of the reinforcing PG. In general, the nanofibrillar objects formed by diffusion of HCl were less homogeneous than those formed with GdL, having shorter fibre lengths by TEM and exhibiting less ‘network-like’ structures by SEM. In addition to being more homogeneous, the gel fibres formed in the presence of GdL also had smaller diameters as determined by TEM than those formed by diffusion of HCl (Fig. S20†), suggestive of their more controlled assembly. This clearly indicates the benefits of assembling DBS-CO_2_H more slowly with a greater degree of control (analogous to slow crystallisation processes). Furthermore, in the case of GdL-induced assembly, there was sometimes evidence of alignment of the self-assembled nanofibers across large regions of the TEM grid (Fig. 9). We do not normally see this effect in TEM samples of these hybrid gels.^17*b*^ It is possible this may result from assembly being a diffusion-induced process, similar to the previous report from Ziemecka *et al.*, however we did not see total alignment or control.^8^

#### Thermal characterisation (*T*_gel_)

The patterned object and outer domain samples from the 0.20 mmol HCl and GdL gels were prepared and placed in closed vials. Samples of DBS-CO_2_H and DBS-CONHNH_2_ were also formed in vials. The *T*_gel_ values for each were determined using the simple, reproducible vial inversion method (Table 2).

**Table 2:** *T*_gel_ values of the patterned gel objects and outer domains for 0.2 mmol HCl and GdL LMWG and hybrid gels, and the single- and multi-component LMWG and hybrid gels, and agarose gel

Gel sample	*T*_gel_	
DBS-CO_2_H only	76 °C	
Agarose-only	93 °C	
	With agarose	No agarose
DBS-CONHNH_2_	94 °C	84 °C
DBS-CONHNH_2_/DBS-CO_2_H	97 °C	94 °C
0.20 mmol HCl, object	>100 °C	>100 °C
0.20 mmol HCl, outer	87 °C	(Liquid)
0.20 mmol GdL, object	>100 °C	>100 °C
0.20 mmol GdL, outer	71 °C	91 °C

For standard samples of DBS-CONHNH_2_ and DBS-CO_2_H (made with GdL), *T*_gel_ values of 84 °C and 76 °C, respectively, were observed. When using GdL, the patterned gel object had *T*_gel_ > 100 °C, consistent with the formation of a multi-component network in which DBS-CO_2_H is assembled in the presence of DBS-CONHNH_2_.^17*b*^ The outer domain had a *T*_gel_ value of 91 °C, consistent with this part of the gel being primarily composed of DBS-CONHNH_2_. In the presence of HCl, the patterned object once again had *T*_gel_ > 100 °C. However, in this case, the outer domain did not behave as a gel, and had more liquid-like characteristics. This suggests that the strongly acidic HCl may be damaging the pre-formed DBS-CONHNH_2_ network, for example through acid-mediated protonation of the acyl hydrazide group or acetal hydrolysis at very low pH values (see further characterisation below). Similar trends in gel thermal stability were observed for gels in the presence of agarose, but in this case, the outer domain in the presence of HCl retained its expected *T*_gel_ value.

#### Rheological characterisation

The mechanical properties of gels can be analysed using oscillatory experiments by parallel plate geometry. The patterned object and outer domain of the HCl and GdL hybrid gels were therefore prepared. To facilitate sample handling and loading onto the rheometer, we focussed on the gels formed in the presence of agarose. A spatula was used to cut and remove the object, including the central hole. A bottomless vial was used to remove a sample of the outer domain. All samples were transferred to the plate by spatula for rheological analysis. It was anticipated that the hole in the patterned object corresponding to the loading reservoir may create variability within rheological measurements. However, we centred the gel object under the rheometer, concordant results were achieved in triplicate, and they are hence reported here (Tables S3–S5 and Fig. S21–S40†).

**Fig. 10:**
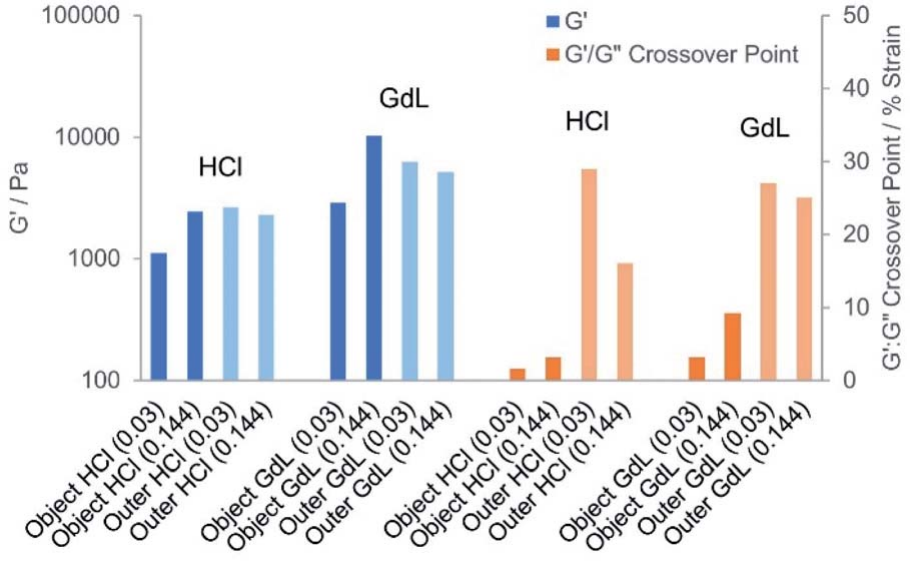
Elastic (*G*′, blue) moduli and *G*′/*G*′′ crossover points (orange) of hydrogels sampled from the patterned object (darker colour) and the outer domains (lighter colour) of gels formed by diffusion of HCl or GdL at different loadings. This shows the stiffening effects on the patterned object of increasing acid loadings, but also shows that the patterned objects are less resistant to strain than the outer domain, presumably as a result of the presence of the cut reservoir.

As the acid concentration increased, stiffer objects were fabricated (Fig. 10 and S41†). For the HCl patterned hybrid objects formed in the presence of agarose, the *G*′ value increased from 1110, to 1780, to 2430 Pa (0.03–0.144 mmol HCl). This increase in stiffness with HCl loading was attributed to a more extensive DBS-CO_2_H network forming as DBS-carboxylate acidification increased. Increasing the acid loading also extended the linear viscoelastic region (LVR) of the HCl hybrid object, with a *G*′/*G*′′ crossover point at a shear strain of 1.6% [0.03 mmol] increasing to 5.4% [0.20 mmol]. However, the outer domain of these gels had a higher *G*′ value of *ca.* 2000–4000 Pa and a LVR that extended significantly beyond 10% strain. In principle, we would have expected the patterned object to have better rheological properties than the outer domain as a result of the additional network.^18^ However, it is important to note that in this experiment the inner domain contains a hole, which will adversely impact on rheological performance, hence although comparisons between different patterned objects have some merit, any comparison between object and outer domain in this system must be made with extreme caution. We suggest the hole is the reason for the slightly weaker than expected performance of the HCl-patterned object.

We found further evidence by rheology that the strong acid may also cause some damage to the supporting DBS-CONHNH_2_ matrix (for example by acetal hydrolysis and/or acyl hydrazide protonation). Indeed, it was noted that the *G*′ value and the resistance to shear strain as indicated by the *G*′/*G*′′ crossover point both decreased in the outer domain in the presence of increasing loadings of HCl.

When using GdL as the acid source, an increase in *G*′ of *ca.* 2500 to 10 000 Pa was observed with increasing GdL loading. The enhanced stiffness achieved with GdL compared with HCl is consistent with the better-defined fibres observed by electron microscopy resulting from a slower, more kinetically-controlled assembly process. This yields a more effective gel network than the rapid acidification and assembly caused by HCl, which will suffer more from inhomogeneities. For the GdL-induced patterned objects, the LVR extended to a maximum of 9%. Comparison with the outer domain (with caution as noted above), indicated that at the higher GdL loadings, the patterned object in this case, actually had a greater stiffness (*G*′ of *ca.* 10 000 Pa instead of *ca.* 5000), as would be expected for the multi-network material, but was significantly less resistant to strain (LVR up to *ca.* 9% strain instead of *ca.* 25% strain). Importantly, in the presence of diffusing GdL, the outer domain retained its rheological performance better than in the presence of diffusing HCl, suggesting enhanced stability of DBS-CONHNH_2_ under the less acidic conditions, and indicating the benefits of using weak acids in these diffusion experiments. Taken as a whole, the results indicate that as GdL diffuses through the gel, protonating DBS-carboxylate and leading to self-assembly of a patterned gel-in-gel DBS-CO_2_H object, this stiffens the material.

## Conclusions

In summary, we have used a double diffusion method from reservoirs cut into a gel in order to create patterned objects with well-defined shapes. Specifically, by diffusing DBS-carboxylate through a pre-formed gel of DBS-CONHNH_2_, and allowing it to be protonated by diffusing acids (HCl or GdL) patterned DBS-COOH objects are created where the diffusion waves overlap. The presence of agarose polymer gel in the system includes physical robustness and facilitates cutting-out of the patterned objects.

The use of HCl, with fast acidification and assembly kinetics, gives rise to sharply-defined objects, but TEM imaging indicates the nanofibers are relatively inhomogeneous and the rheological performance is fairly poor. Furthermore, there is some evidence that the strong acid damages the supporting DBS-CONHNH_2_ network. Using GdL, with slower acidification and assembly kinetics gives rise to slightly larger, less tightly defined objects, but they have significantly enhanced nanofibrillar character, greater rheological robustness, and the overall system appears to have greater stability to the milder pH regime. In general, increasing the loading of either acid gave rise to stiffer objects that were better spatially defined.

The shape of the patterned gel-in-gel object is an emergent property programmed by both the shape of the central reservoir and the geometry of the external reservoirs. In general, when using HCl, which achieves rapid assembly, or when using lots of external reservoirs (four or more), the shape of the central reservoir became dominant in controlling the pattern of the shaped gel-in-gel object. However, when using GdL which only slowly releases H^+^, or when using a limited number of external reservoirs (*e.g.* two or three), the geometry of those external reservoirs relative to the central reservoir becomes dominant on the shape of the patterned object. These emergent properties demonstrate how multiple factors in combination can achieve more subtle control over gel patterning than previously reported.

We suggest that this approach should be applicable to many other pH-triggered gels, and may be suitable for creating patterned gels for tissue engineering.^4,19^ If done in advance of tissue growth, it would be essential to return the pH to a biologically acceptable level. If done during tissue growth as a dynamic process, it would be important to ensure the use of weak acids and/or gelators with carefully chosen p*K*_a_ values such that the triggering process could be tolerated by cells. In this regard, it would also be of great value to create systems which undergo self-assembly in response to simple diffusing triggers other than acids. It is also suggested that injecting diffusing triggers into a self-assembled gel may be preferable to the more disruptive effect of cutting reservoirs – work in these directions is currently in progress in our laboratories.

## Data availability

We have provided data in the ESI† (the key data is photographs, rheology and excel graphs etc - all included).

## Author contributions

The manuscript was written through contributions of all authors. HSC performed most of the experimental work, with guidance and supervision from CCP. LS performed some of the preliminary studies on this system. DKS envisioned and managed the project and coordinated manuscript writing.

## Conflicts of interest

There are no conflicts to declare.

## Supplementary Material

SC-012-D1SC03155D-s001
