# Supplementary material for: Double diffusion for the programmable spatiotemporal patterning of multi-domain supramolecular gels
Source: Chem Sci. 2021 Aug 18;12(36):12156–64. doi: 10.1039/d1sc03155d (PMC8457394; doi:10.1039/d1sc03155d)
Supplement: SC-012-D1SC03155D-s001 [file SC-012-D1SC03155D-s001.pdf]

Electronic Supplementary Material (ESI) for Chemical Science.
This journal is © The Royal Society of Chemistry 2021

Double diffusion for the programmable spatio-temporal patterning of multi-domain supramolecular gels

Hannah S. Cooke,[a] Lisa Schlichter,[a] Carmen C. Piras[a] and David K. Smith[a] *

a: Department of Chemistry, University of York, Heslington, York, YO10 5DD, UK.

Email: david.smith@york.ac.uk

SUPPORTING INFORMATION

Contents

S1. General Experimental Methods
S2. Gel Preparation
S3. NMR Studies
S4. Infrared (IR) Spectroscopy
S5. Transmission and Scanning Electron Microscopy (TEM and SEM)
S6. Thermal Studies (T_{gel})
S7. Rheology
S8. References

S1. General Experimental Methods

All compounds used in synthesis and analysis were purchased from standard commercial suppliers and used as received. The synthesis of DBS-CONHNH$_2$ and DBS-COOH were performed in good yields applying previously reported methods.[1,2] ^{1}H NMR spectra were recorded using a Jeol 400 spectrometer (^{1}H 400 MHz) or a 500 spectrometer (^{1}H 500 MHz). Samples were prepared in D_2O and chemical shifts (δ) are reported in parts per million (ppm). IR spectra of xerogels were recorded on a PerkinElmer Spectrum Two FT-IR spectrometer. TEM images were obtained on a FEI Tecnai 12 G^2 fitted with a CCD camera. Fibre sizes were measured using the *ImageJ* software. SEM images were taken using a JEOL JSM-7600F field emission SEM. Rheology was measured on a Malvern Instruments Kinexus Pro+ Rheometer fitted with a 2 cm parallel plate geometry.

S2. Gel Preparation

S2.1 Preparation of gels in trays for RD-SA

A suspension of DBS-CONHNH$_2$ (8.43 x 10^{-3} mmol, 0.4 % wt/vol) in water (5 mL) was prepared, and Thymol Blue (20 μL, 1% in EtOH) was added. For the gels with agarose, agarose (1.0 % wt/vol) was added to the suspension of DBS-CONHNH$_2$ and Thymol Blue before sonication. The opaque yellow solution was sonicated for 15 minutes, then heated with a heat gun until complete dissolution of the compound. The hot solution was transferred to a 5 cm x 5 cm gel tray and left to cool overnight. Circular holes were then cut into the gel using a pipette tip, in either a square or triangular arrangement.

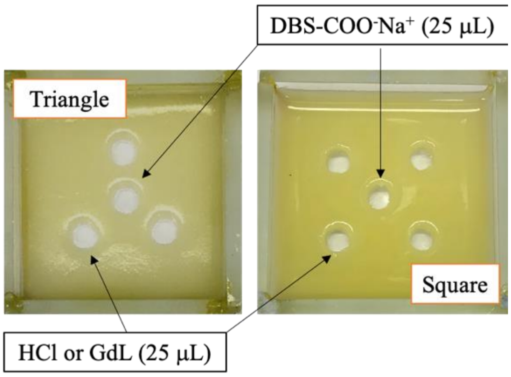

Fig. S1. Gel trays prior to loading with DBS-Carboxylate and acids with (left) triangular and (right) square loading geometries.

A basic solution of DBS-CO$_2$H (25 μL, 8 mg/60 μL 1M NaOH) was added to the central hole. The outer holes were loaded with HCl or GdL (25 μL per hole) of total acid loading 0.03 mmol, 0.06, 0.144, or 0.20 mmol. The loading of each HCl/GdL hole was the total acid loading divided by the number of outer holes [for 0.03 mmol $HCl_{(Triangle)}$, 0.01 mmol HCl, 25 μL per hole x 3]. After 28 hours, the multi-component hydrogel object was fabricated. Using a spatula, the object was cut out and removed from the surrounding gel to yield the free-standing object.

The procedure was repeated as described above to fabricate the more intricately shaped hydrogel objects but using different shaped cutters for the holes. The more complex patterns were studied with the 0.20 mmol HCl or GdL hybrid gels.

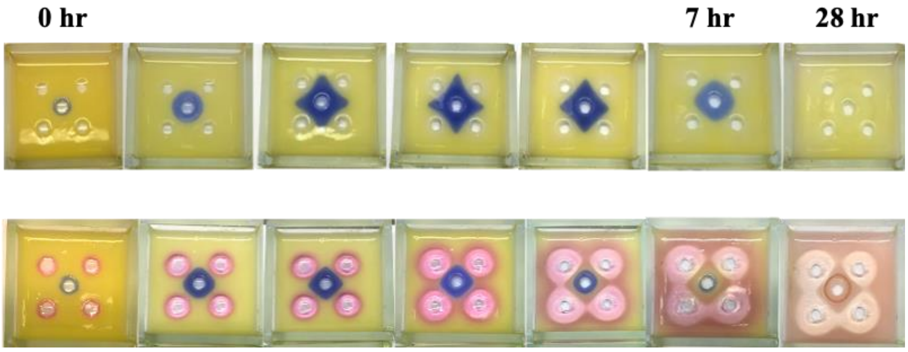

Figure S2. The diffusion of the DBS-CO_2H gelator precursors for the 0.06 mmol $GdL_{(Square)}$ (upper) and $HCl_{(Square)}$ (lower) hybrid gels. The basic DBS-COO^- solution is indicated blue by TB, the acidic HCl, pink. GdL, of a higher pH, maintained the yellow colour of the gel. Diffusion monitored for 7 hrs, and then at 28 hrs. Complete acidification was observed after 28 hours of diffusion.

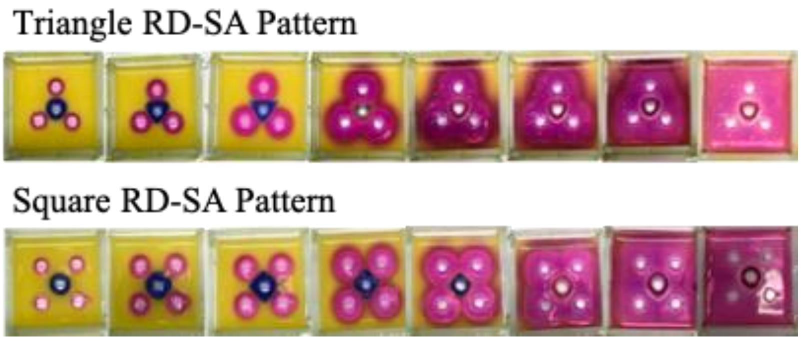

Figure S3. The triangle and square gel patterns, for 0.144 mmol $HCl_{(Triangle)}$ (upper), and 0.20 mmol $HCl_{(Square)}$ (lower) hybrid gels. The basic DBS-COO^- solution is indicated blue by TB; the acidic HCl, pink.

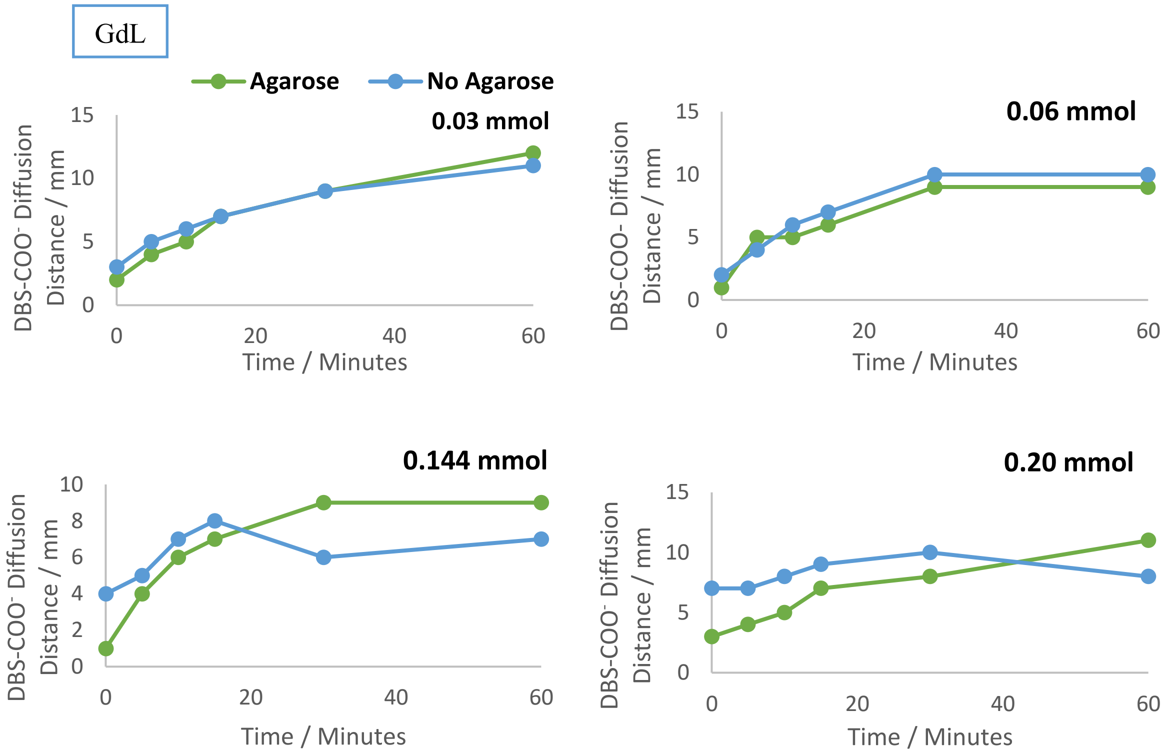

Figure S4. The distance of blue DBS-COO$^-$ solution diffusion from the central loaded hole, relative to the 5 cm tray, for the first hour of diffusion (60 min). The graphs compare the GdL$_{(Square)}$ LMWG (no agarose, blue line) and hybrid (agarose, green line) gels, at each loading.

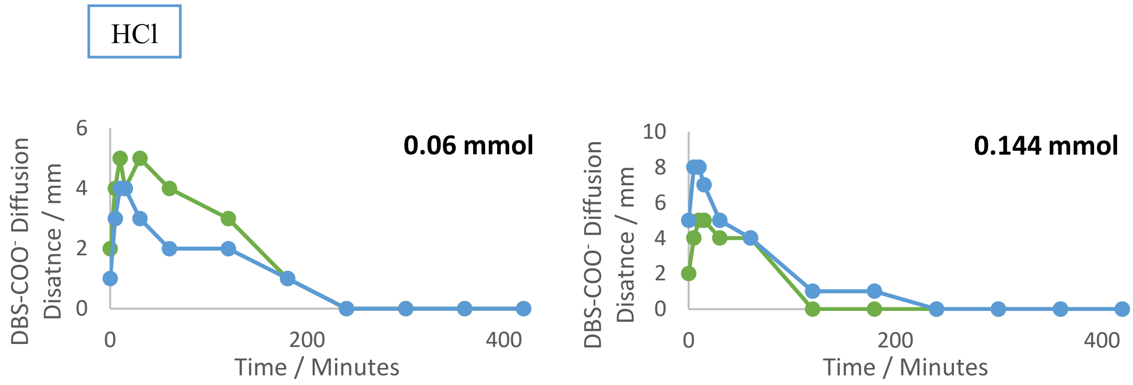

Figure S5. The distance of blue DBS-COO$^-$ solution diffusion from the central loaded hole, relative to the 5 cm tray, over a period of 7 hours (420 min). The graphs compare the HCl$_{(Square)}$ LMWG (no agarose, blue line) and hybrid (agarose, green line) gels, for 0.06 and 0.144 mmol HCl.

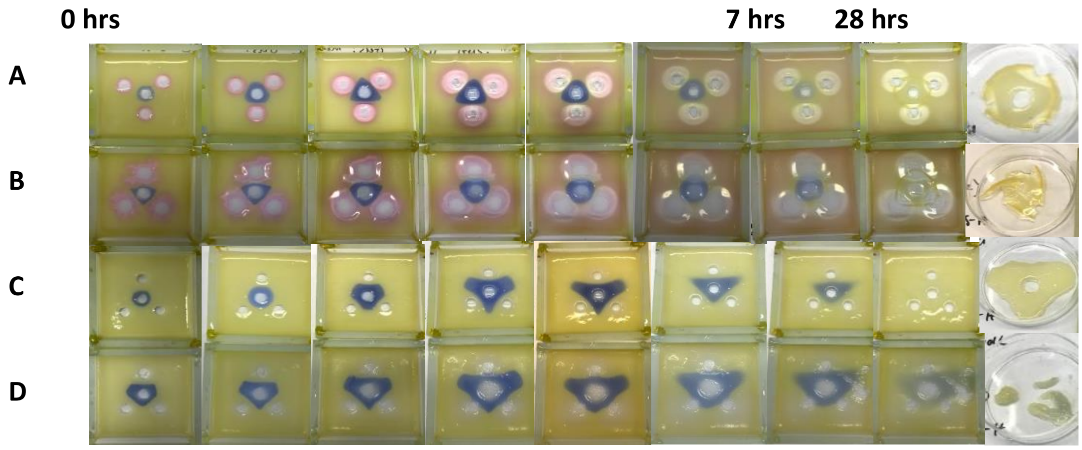

Figure S6. Gel object formation for the 0.03 mmol $HCl_{(Triangle)}$ hybrid (A) and LMWG (B), $GdL_{(Triangle)}$ hybrid (C), and LMWG (D) gels, pictured between 0 and 7 hours, and then at 28 hours. The structure was cut and removed to give the free-standing object.

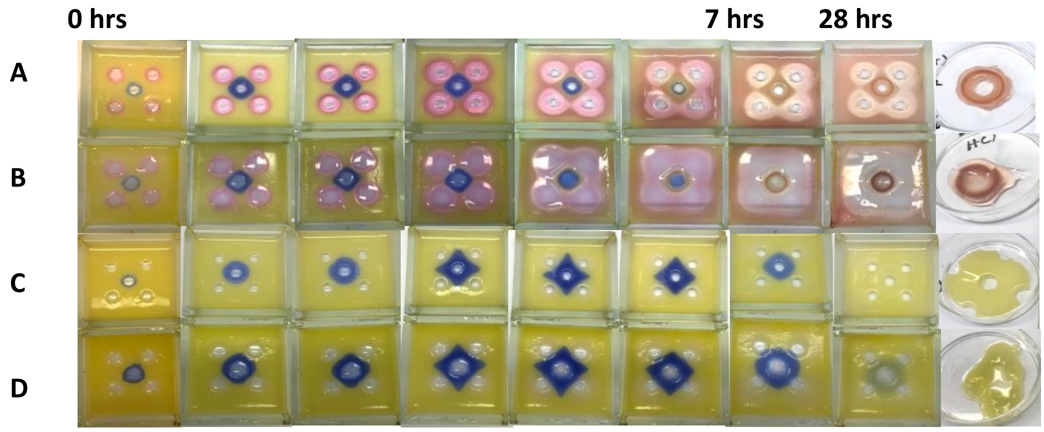

Figure S7. Gel object formation for the 0.06 mmol $HCl_{(Square)}$ hybrid (A) and LMWG (B), $GdL_{(Square)}$ hybrid (C), and LMWG (D) gels, pictured between 0 and 7 hours, and then at 28 hours. The structure was cut and removed to give the free-standing object.

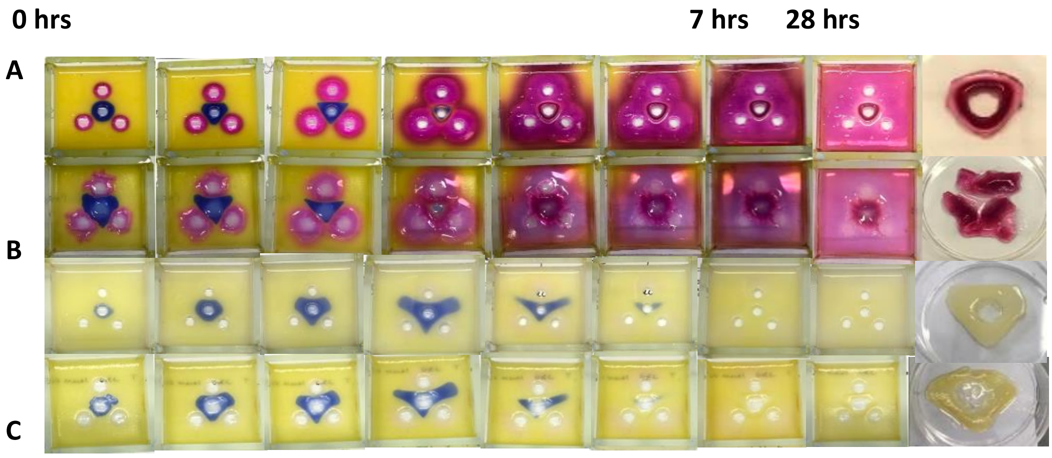

Figure S8. Gel object formation for the 0.144 mmol $HCl_{(Triangle)}$ hybrid (A) and LMWG (B), $GdL_{(Triangle)}$ hybrid (C), and LMWG (D) gels, pictured between 0 and 7 hours, and then at 28 hours. The structure was cut and removed to give the free-standing object.

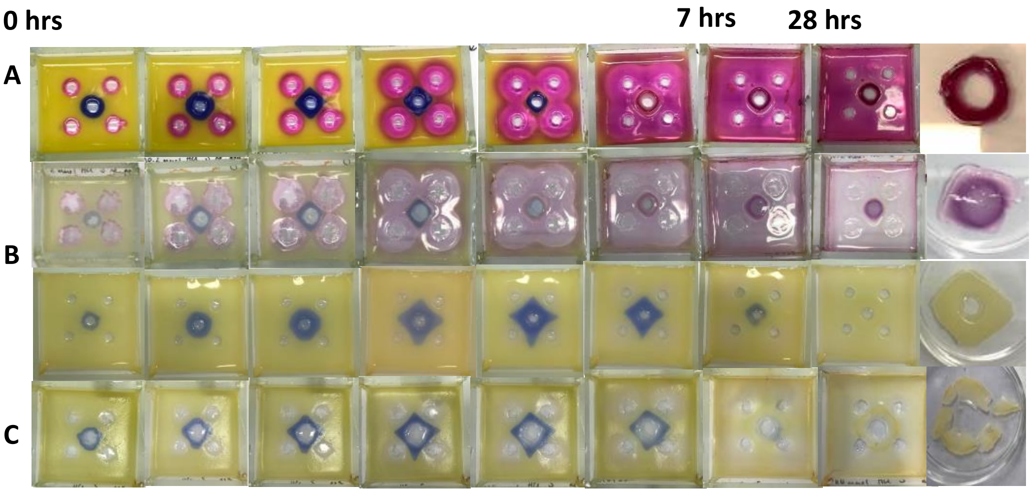

Figure S9. Gel object formation for the 0.20 mmol $HCl_{(Square)}$ hybrid (A) and LMWG (B), $GdL_{(Square)}$ hybrid (C), and LMWG (D) gels, pictured between 0 and 7 hours, and then at 28 hours. The structure was cut and removed to give the free-standing object.

S3. NMR Studies

Gel objects were studied by NMR: HCl and GdL, each at 0.03, 0.06, 0.144 and 0.20 mmol loading for both the LMWG and hybrid gels and in the triangle and square patterning arrangement of precursor holes.

Gel objects were prepared in 5 mL gel trays but in the absence of Thymol Blue indicator. Using a spatula, the object was cut out and removed from the surrounding gel, placed in a vial and left to dehydrate to form the xerogel. The object xerogel was dissolved in DMSO-d_6 solvent (0.71 mL) and sonicated until complete dissolution of the xerogel. Acetonitrile (2 μL, δ = 2.09 ppm) was added as an internal standard, and the solution filtered and transferred into an NMR tube.

The quantity of LMWGs in the object was therefore calculated by using the peak integration of the LMWG aromatic peak (*ca.* 7 – 8 ppm) relative to that of acetonitrile.

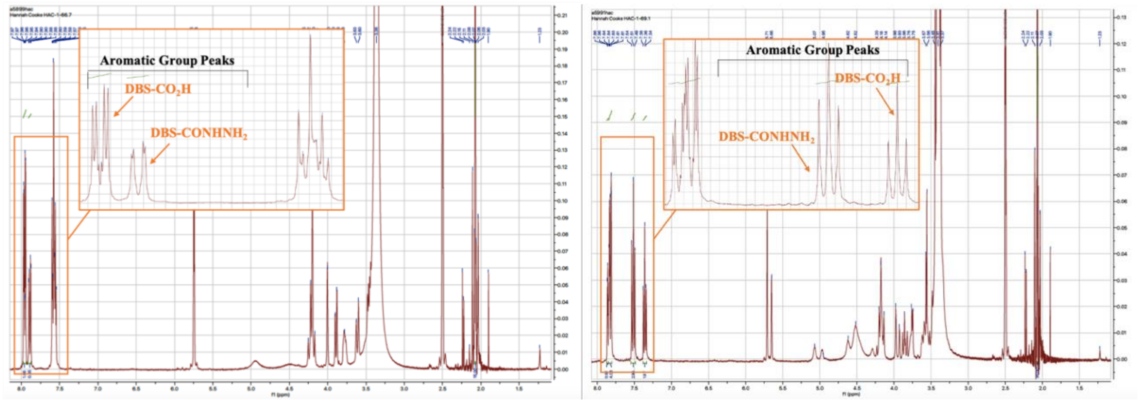

Figure S10. ^{1}H NMR spectra of 0.03 mmol $HCl_{(Square)}$ (left) and $GdL_{(Square)}$ (right) LMWG xerogel object. The enlarged regions highlight the aromatic gelator peaks used to obtain the relative integrals.

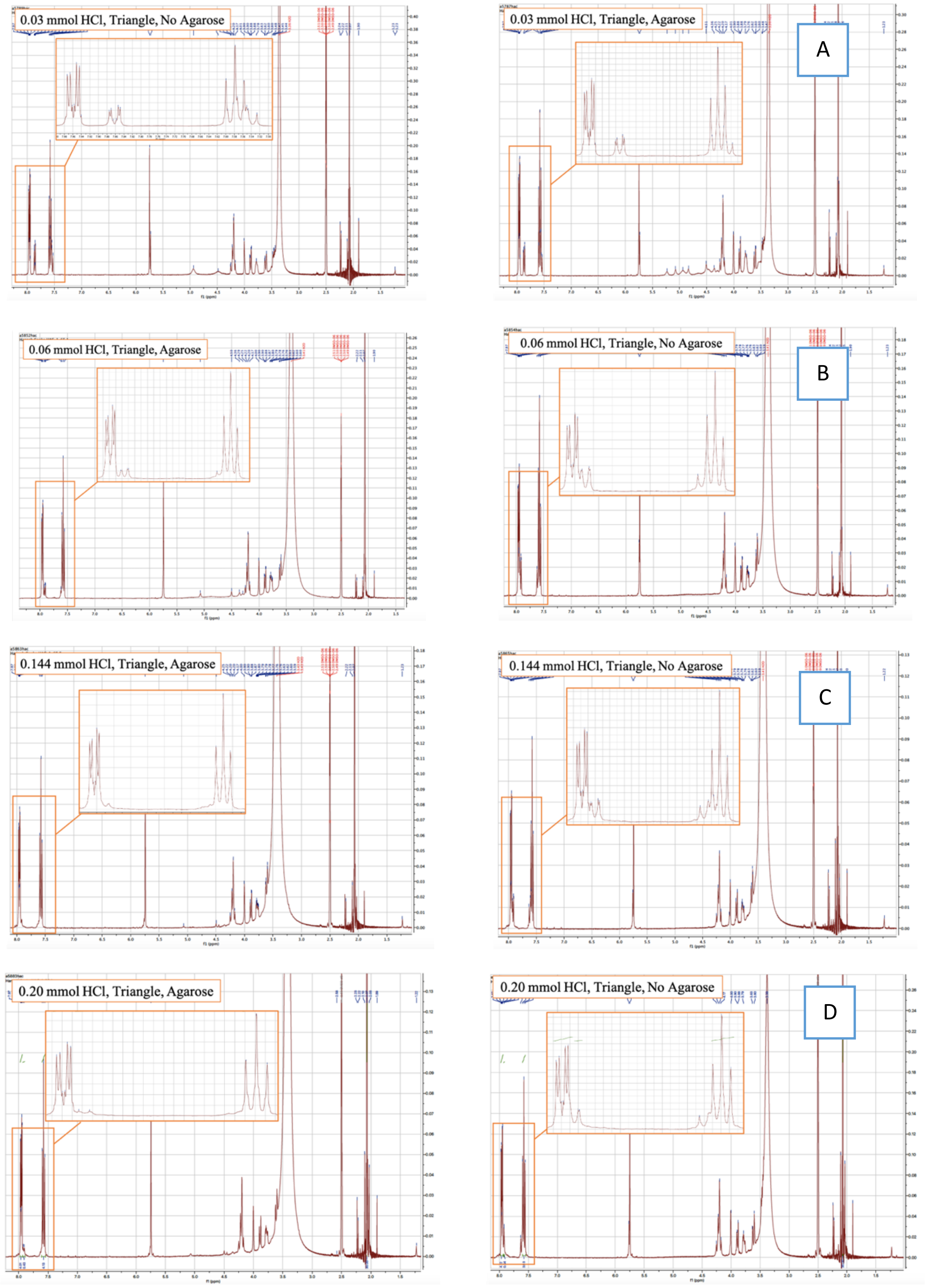

Figure S11. ^{1}H NMR spectra for $HCl_{(Triangle)}$ at (A) 0.03, (B) 0.06, (C) 0.144, and (D) 0.20 mmol HCl, hybrid (Agarose, left) and LMWG (No Agarose, right) gels.

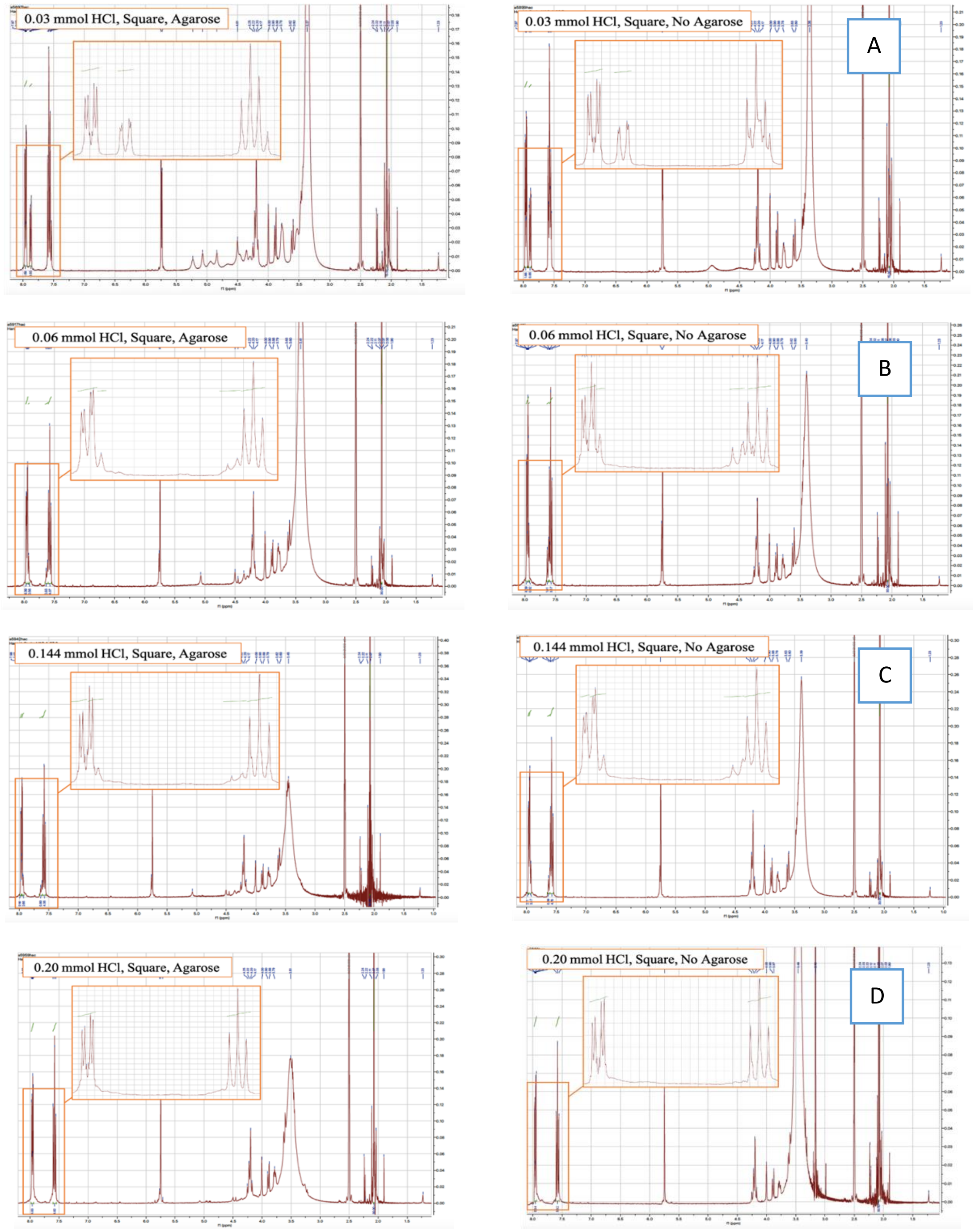

Figure S12. ^{1}H NMR spectra for $HCl_{(Square)}$ at (A) 0.03, (B) 0.06, (C) 0.144, and (D) 0.20 mmol HCl, hybrid (Agarose, left) and LMWG (No Agarose, right) gels.

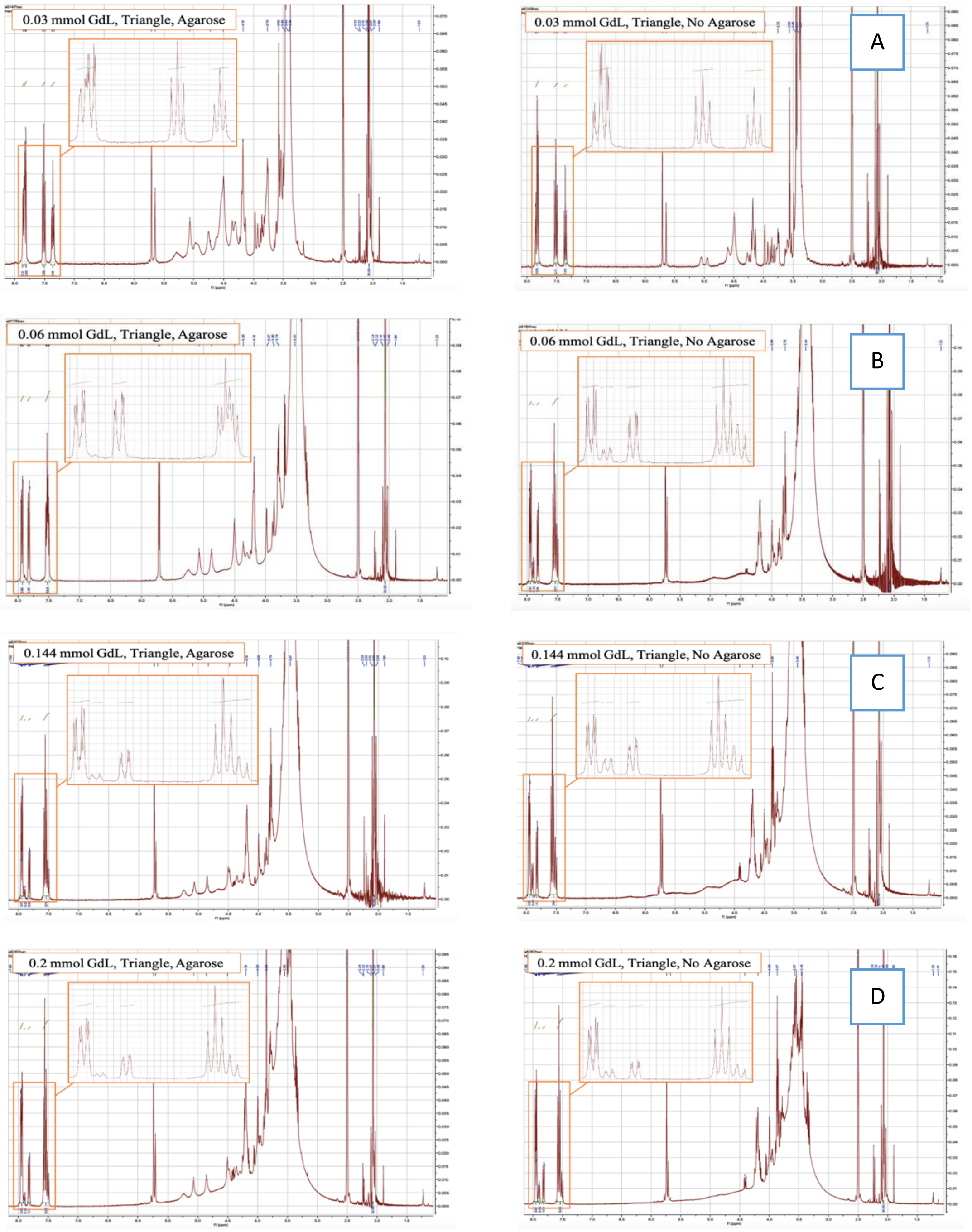

Figure S13. ^{1}H NMR spectra for GdL$_{(Triangle)}$ at (A) 0.03, (B) 0.06, (C) 0.144, and (D) 0.20 mmol HCl, hybrid (Agarose, left) and LMWG (No Agarose, right) gels.

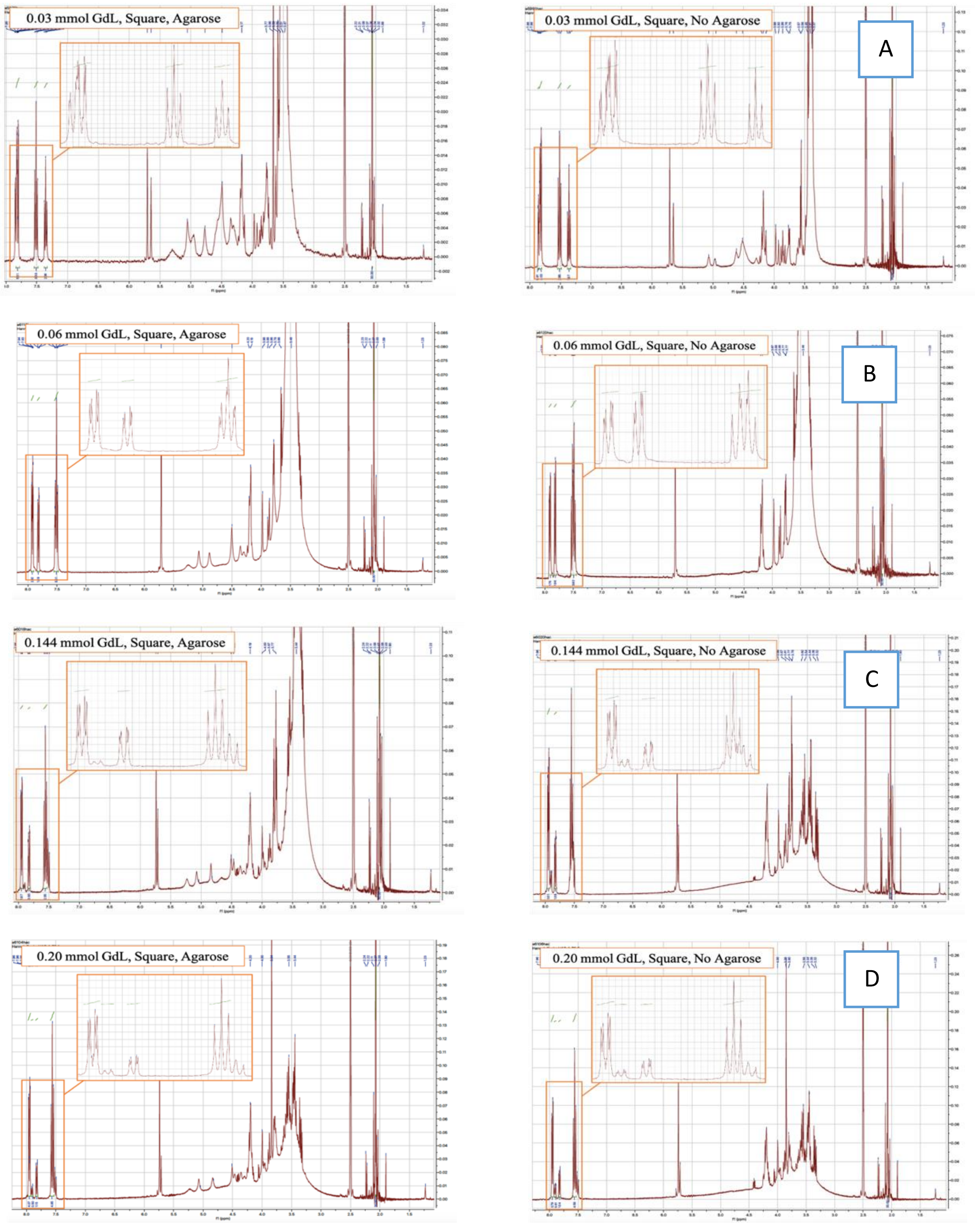

Figure S14. ^{1}H NMR spectra for $GdL_{(Square)}$ at (A) 0.03, (B) 0.06, (C) 0.144, and (D) 0.20 mmol HCl, hybrid (Agarose, left) and LMWG (No Agarose, right) gels.

The quantity of LMWGs in the object was calculated by using the peak integration of the LMWG aromatic peak (δ_H ca. 7 – 9 ppm, 4H) relative to that of acetonitrile (δ_H = 2.07 ppm, 3H). The integral of acetonitrile was multipled by 10 and set equal to 30, hence the LMWG integral was divided by 40 rather than 4 (corresponding to the 4H). The moles of acetonitrile added (2 μL) were 0.0383 mmol [0.786 g mL^{-1}, 41.05 g mol^{-1}]. The moles of LMWG in the object was calculated as follows:

$$moles\ of\ LMWG\ in\ object\ (mmol) = \frac{Integral\ of\ LMWG\ peak}{40} \times\ 0.0383\ mmol$$

The percentage of DBS-CO_2H within the object was then calculated. The total mass of DBS-CO_2H LMWG [446.41 g mol^{-1}] added to the central hole was 3.33 mg (25 µL, 8 mg/60 µL 1M NaOH).

$$mass\ of\ DBS\_CO_2H\ in\ object\ (mg) = moles\ of\ DBS\_CO_2H\ in\ object\ (mmol) \times\ 446.41\ g\ mol^{-1}$$

$$\%\ of\ DBS_{CO_2H}LMWG\ in\ object = \frac{mass\ of\ DBS_{CO_2H}in\ object\ (mg)}{3.33\ mg} \times\ 100$$

Table S1: Percentage of DBS-CO_2H LMWG in the xerogel object for $HCl_{(Square)}$, $HCl_{(Triangle)}$, $GdL_{(Square)}$, and $GdL_{(Triangle)}$ LMWG gels.

Acid Loading / mmol	Percentage of DBS-CO_2H LMWG (%) in xerogel object			
	$HCl_{(Triangle)}$	$HCl_{(Square)}$	$GdL_{(Triangle)}$	$GdL_{(Square)}$
0.03	51.73	21.31	17.07	24.00
0.06	58.28	45.82	25.67	22.59
0.144	48.26	61.36	26.31	45.05
0.20	52.88	74.96	50.19	48.65

S4. Infrared (IR) Spectroscopy

The single- and multi-component LMWG gels were made in the absence of Thymol Blue. The LMWG and hybrid object and outer domains of the $HCl_{(Square)}$ system, at 0.03, 0.06, 0.144, and 0.20 mmol HCl loadings were analysed. Using a spatula, the object and outer domain were cut out and removed from the surrounding gel, placed in a vial and left to dehydrate to form xerogels. The xerogels were crushed, and the powder was placed into the infrared spectrophotometer and the spectra recorded.

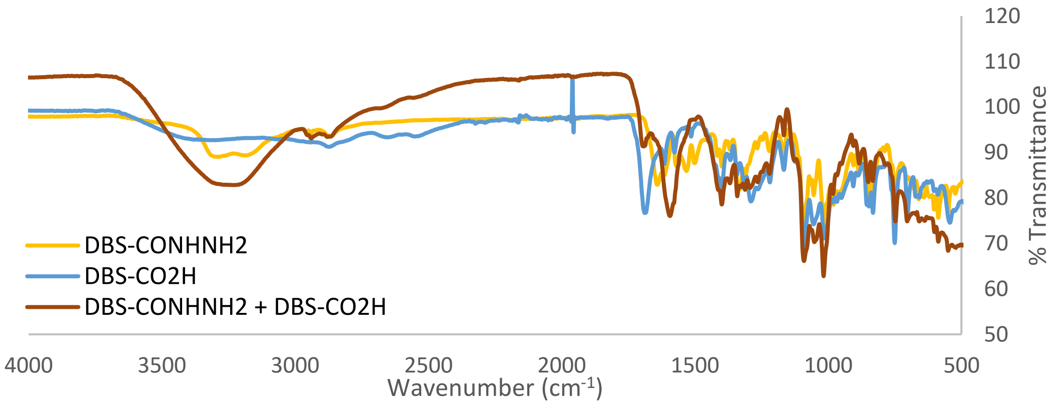

Figure S15. IR spectra of DBS-$CONHNH_2$ (0.4% wt/vol, yellow line) and DBS-CO_2H (0.4% wt/vol, blue line), DBS-$CONHNH_2$/DBS-CO_2H (0.2% wt/vol of each LMWG, red line) LMWG xerogels.

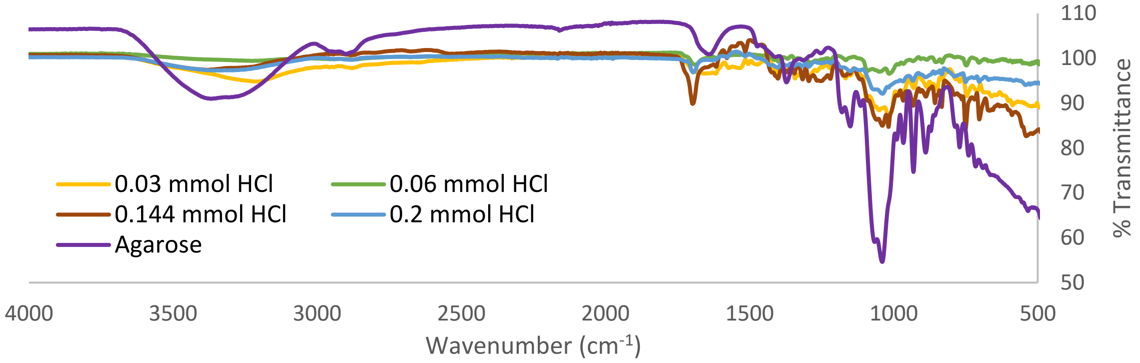

Figure S16. IR spectra of hybrid xerogel objects at 0.03 mmol (yellow line), 0.06 mmol (green line), 0.144 mmol (red line), and 0.20 mmol (blue line) HCl loadings; and agarose (purple line).

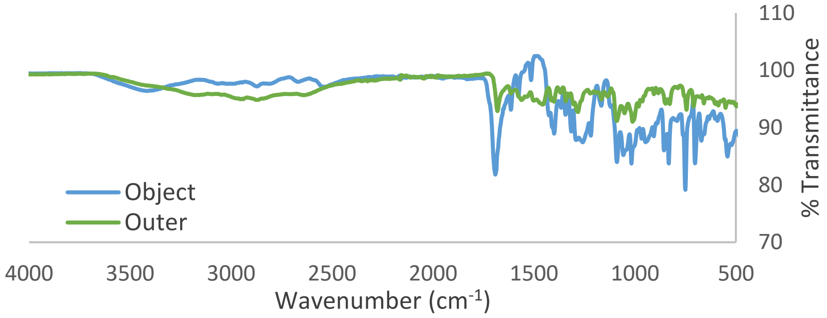

Figure S17. IR spectra of the object (blue line) and outer domain (green line) domains of the 0.144 mmol $HCl_{(Square)}$ LMWG xerogel.

Table S2. IR spectroscopic shifts of xerogels of the single and multi-component LMWG.

**Spectroscopic Signal**	**Wavenumber / cm^{-1}**				
	**DBS-CONHNH$_2$**		**DBS-CONHNH$_2$/DBS-CO$_2$H**		**DBS-CO$_2$H**
	**Agarose**	**No Agarose**	**Agarose**	**No Agarose**	**No Agarose**
**C=O**	1635	1640	1664	1695	1687
**N-H**	3314	3265	3246	3216	-

S5. Transmission and Scanning Electron Microscopy (TEM and SEM)

The object and outer domains from the 0.20 mmol $HCl_{(Square)}$ and $GdL_{(Square)}$ LMWG and hybrid gels, were studied by TEM and SEM. The gels were prepared in 5 mL gel trays but in the absence of Thymol Blue indicator. Using a spatula, the object and outer domain were cut out and removed from the surrounding gel and covered with water to prevent dehydration.

TEM Sample Preparation. The TEM gel samples were affixed to HT-treated formvar/carbon 200 mesh copper grids then negatively stained with 1% aqueous uranyl acetate. Images were taken between x 6800 and x 49000 magnification.

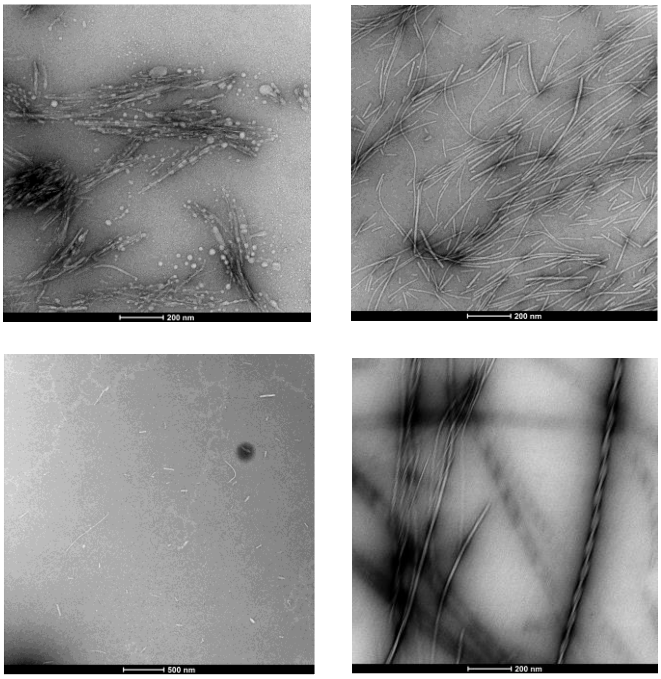

Figure S18. TEM images for the (top left) HCl, scale bar 200 nm, (top right) GdL hybrid objects, scale bar 200 nm; (bottom left) HCl hybrid outer domains scale bar 500 nm; (bottom right) GdL LMWG outer domain, scale bar 200 nm.

SEM Preparation. Samples were frozen in liquid nitrogen slush and freeze-dried at -60 °C with Polaron E5380 freeze-drier. Samples were affixed to SEM stubs and sputter coated with 5nm of gold/palladium on a Polaron SC7640 sputter coater. Images were taken at x 10000 and x 20000 magnification.

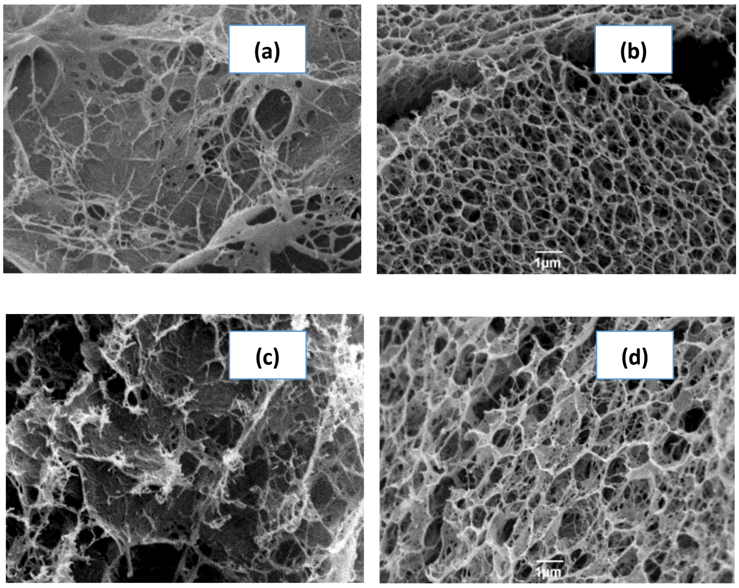

Figure S19. SEM images: 0.20 mmol GdL (a) LMWG and (b) hybrid object; 0.2 mmol HCl (c) LMWG and (d) hybrid object. Scale bar: 1 µm.

Fibre Width Measurements. Using the software ImageJ, fibre widths were measured from the TEM images obtained. For each sample, *ca*. 50-100 fibres were measured, and the percentage of fibres of width within the specified ranges calculated.

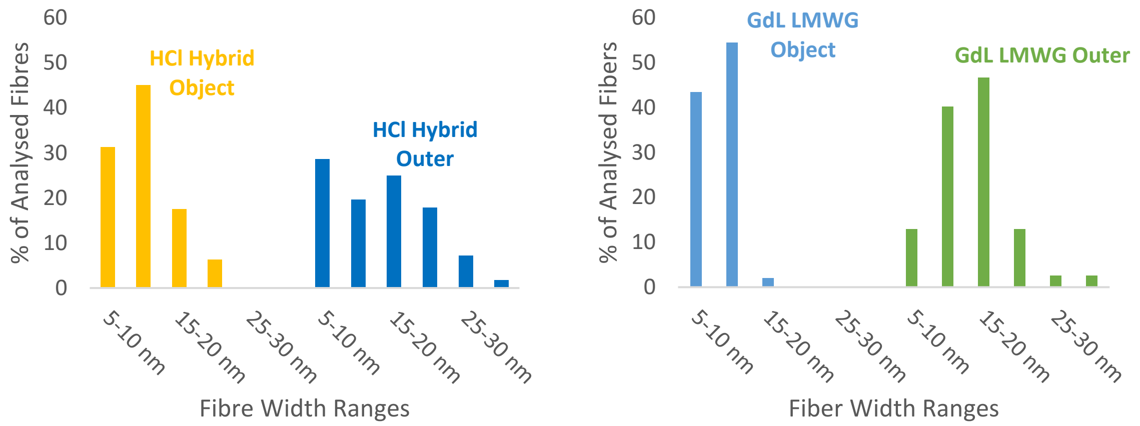

Figure S20. Gelator fibre width size ranges for 0.20 mmol HCl (left) and 0.20 mmol GdL (right) hybrid gel objects and outer domains.

S6. Thermal Studies (T_{gel})

The T_{gel} values of the object and outer domains from the 0.20 mmol $HCl_{(Square)}$ LMWG and hybrid gel were obtained. The gels were prepared in 5 mL trays but in the absence of Thymol Blue indicator. Using a spatula, the object and outer domain were cut out and removed from the surrounding gel and placed into a closed vial.

T_{gel} values for the single-component LMWG gels of DBS-$CONHNH_2$, DBS-CO_2H, agarose, and the multi-component gel of DBS-$CONHNH_2$/DBS-CO_2H were obtained. T_{gel} values for the hybrid single-component gel of DBS-$CONHNH_2$, and the multi-component DBS-$CONHNH_2$/DBS-CO_2H were also obtained. These gels were made in vials.

The vials were placed in a thermo-controlled oil bath, with an initial temperature of 30 °C, programmed to rise to a temperature of 100 °C. At each increase of 1 °C, vials were removed from the oil bath, and tipped sideways for the object and outer gel samples or inverted for the vial-filling LMWG and hybrid gels. For the objects and outers, the T_{gel} value recorded was the temperature at which the sample began to melt, indicated by a gel-sol transition. The temperature at which the gels were no longer self-supporting. It should be noted that while the gels in vials are sample-spanning, this is not necessarily the case for the cut out gels, as such some caution must be applied in comparing T_{gel} values between the two different methods.

S7. Rheology

Rheological studies were conducted on the object and outer domains from the $HCl_{(Square)}$ LMWG gels, and $HCl_{(Square)}$ and $GdL_{(Square)}$ hybrid gels, at 0.03, 0.06, 0.144, and 0.20 mmol acid loadings. The gels were prepared in 5 mL trays, but in the absence of Thymol Blue indicator. The object was cut and removed from the surrounding gel using a spatula. A bottomless vial was used to cut the outer gel domain, in order to obtain a sample of appropriate gel dimensions. Samples were covered with water to prevent dehydration. Measurements were carried out at 25 °C using a 2 cm parallel plate geometry and a gap of 1.7 mm. Amplitude

sweep experiments were performed in the range of 0.01-100% shear strain at a 1 Hz frequency to identify the linear viscoelastic region (LVR). Frequency sweep experiments were performed between 0.1 and 100 Hz using a shear strain of 0.01%. To ensure reproducibility, the reported value is the average value of duplicate or triplicate concordant results – the rheology was often challenging, particularly in the case of objects containing holes.

Table S3. Elastic (G') and viscous (G'') moduli, and % shear strain of object and outer domains for the $HCl_{(Square)}$ hybrid gels, at 0.03, 0.06, 0.144, and 0.20 mmol HCl. All were gels, with G' > G''.

Gel Domain	HCl Loading	Elastic Modulus (G')	Viscous Modulus (G'')	% Shear Strain
**Object**	0.03 mmol	1110	113	1.59
	0.06 mmol	1780	124	2.52
	0.144 mmol	2430	757	3.169
	0.20 mmol	1980	939	5.38
**Outer**	0.03 mmol	2650	118	29.2
	0.06 mmol	4350	158	18.2
	0.144 mmol	2280	104	15.9
	0.20 mmol	3870	169	10.8

Table S4. Elastic (G') and viscous (G'') moduli, and % shear strain of object domain for the $HCl_{(Square)}$ LMWG gels, at 0.03, 0.06, 0.144, and 0.20 mmol HCl. All were gels, with G' > G'', except 0.144 mmol HCl where G' < G''; the object appeared gel-phase upon formation and loading. The outer domains exhibited a gel-sol transition during RD-SA with HCl, therefore could not be analysed by rheology.

Gel Domain	HCl Loading	Elastic Modulus (G')	Viscous Modulus (G'')	% Shear Strain
**Object**	0.03 mmol	488	438	5.02
	0.06 mmol	1980	846	7.94555
	0.144 mmol	101	324	7.947
	0.20 mmol	824	80.2	6.47

Table S5. Elastic (G') and viscous (G'') moduli, and % shear strain of object and outer domains for the $GdL_{(Square)}$ hybrid gels, at 0.03, 0.06, 0.144, and 0.20 mmol GdL. All were gels, with G' > G''.

Gel Domain	GdL Loading	Elastic Modulus (G')	Viscous Modulus (G'')	% Shear Strain
**Object**	0.03 mmol	2860	968	3.193
	0.06 mmol	2270	129	3.12
	0.144 mmol	10300	660	9.195
	0.20 mmol	10400	776	4.68
**Outer**	0.03 mmol	6210	783	26.9
	0.06 mmol	4230	294	20.047
	0.144 mmol	5150	239	25
	0.20 mmol	3700	239	26.6

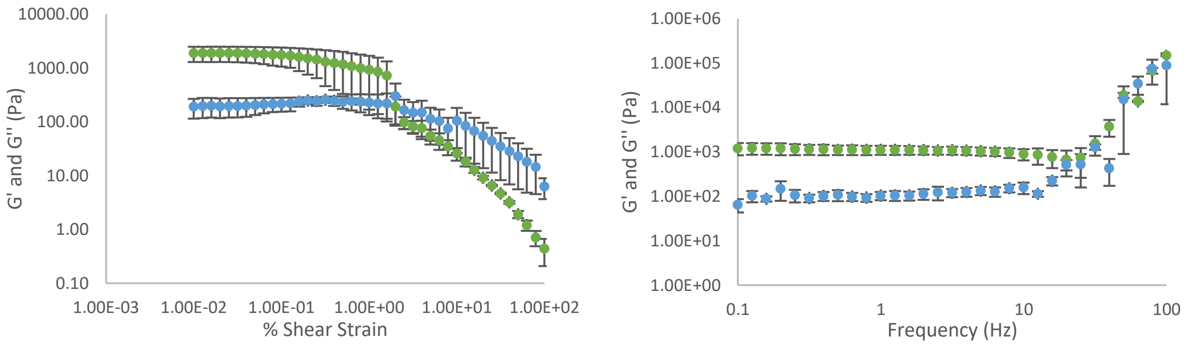

Figure S21. Elastic (G’, green circles) and viscous (G’’, blue circles) moduli of the 0.03 mmol $HCl_{(Square)}$ hybrid object, with increasing shear strain (left) and frequency (right).

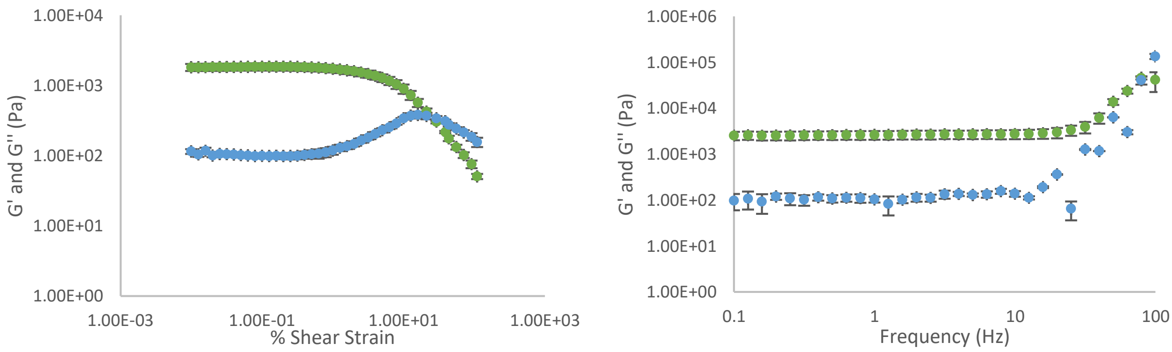

Figure S22. Elastic (G’, green line) and viscous (G’’, blue line) moduli of the 0.03 mmol $HCl_{(Square)}$ hybrid outer, with increasing shear strain (left) and frequency (right).

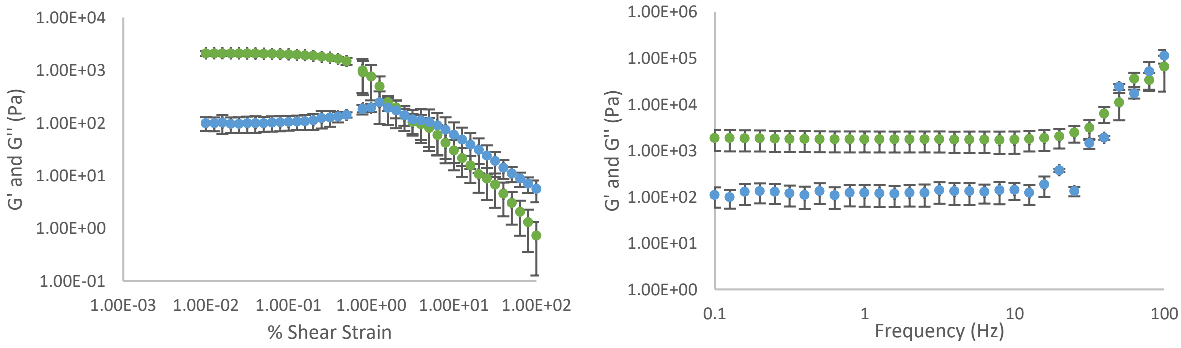

Figure S23. Elastic (G’, green line) and viscous (G’’, blue line) moduli of the 0.06 mmol $HCl_{(Square)}$ hybrid object, with increasing shear strain (left) and frequency (right).

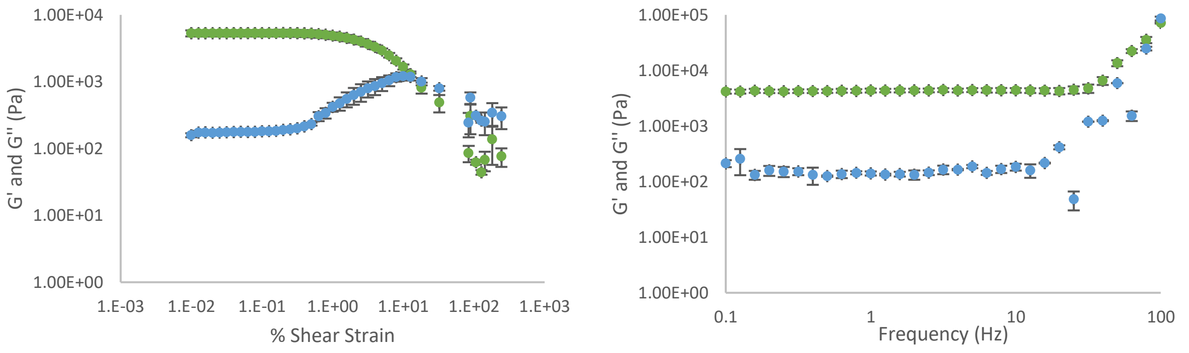

Figure S24. Elastic (G', green line) and viscous (G'', blue line) moduli of the 0.06 mmol $HCl_{(Square)}$ hybrid outer, with increasing shear strain (left) and frequency (right).

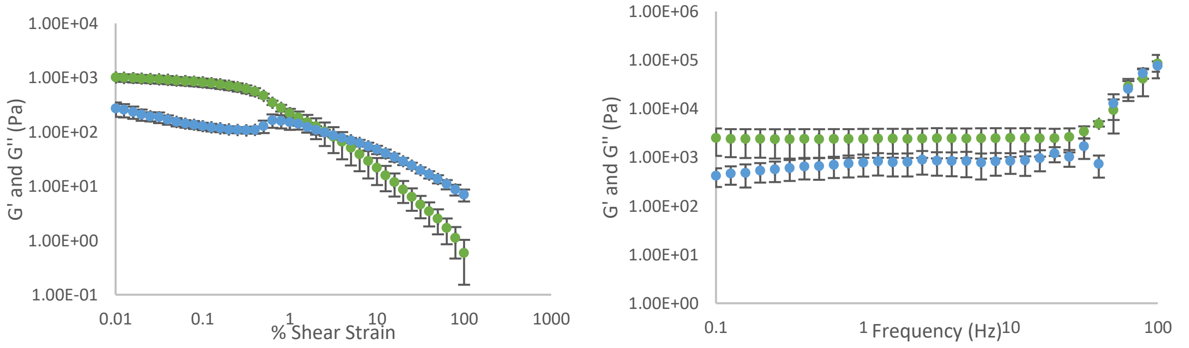

Figure S25. Elastic (G', green line) and viscous (G'', blue line) moduli of the 0.144 mmol $HCl_{(Square)}$ hybrid object, with increasing shear strain (left) and frequency (right).

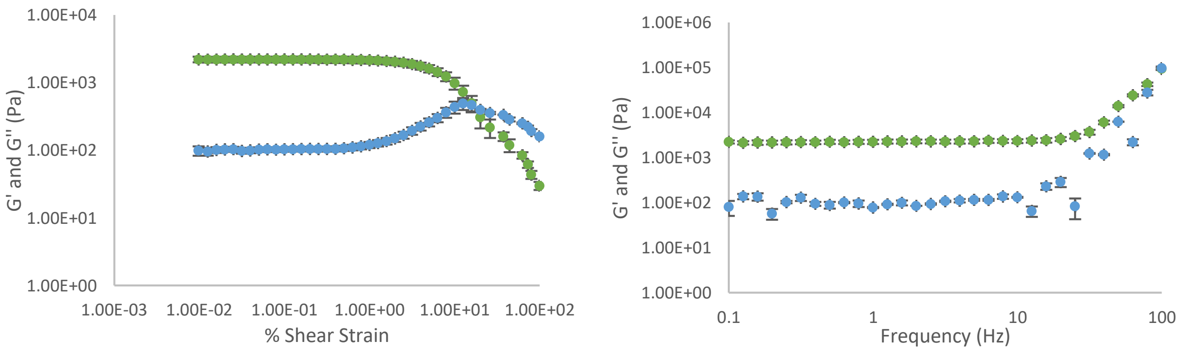

Figure S26. Elastic (G', green line) and viscous (G'', blue line) moduli of the 0.144 mmol $HCl_{(Square)}$ hybrid outer, with increasing shear strain (left) and frequency (right).

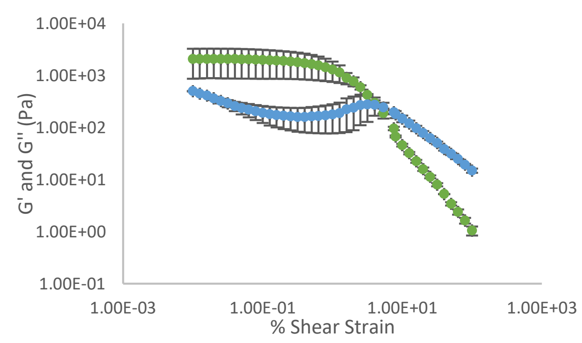

Figure S27. Elastic (G’, green line) and viscous (G’’, blue line) moduli of the 0.20 mmol $HCl_{(Square)}$ hybrid object, with increasing shear strain (left) and frequency (right).

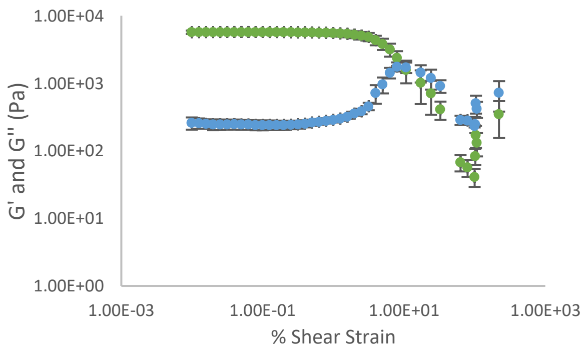

Figure S28. Elastic (G’, green line) and viscous (G’’, blue line) moduli of the 0.20 mmol $HCl_{(Square)}$ hybrid outer, with increasing shear strain (left) and frequency (right).

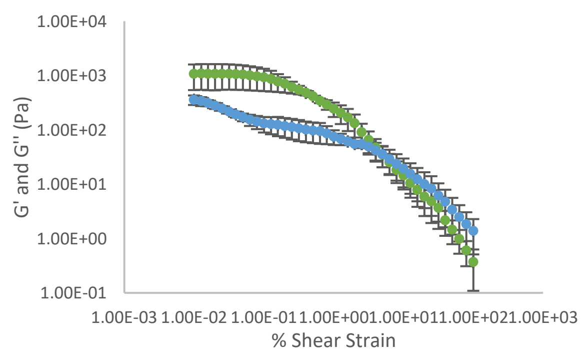

Figure S29: Elastic (G’, green line) and viscous (G’’, blue line) moduli of the 0.03 mmol $HCl_{(Square)}$ LMWG object, with increasing shear strain (left) and frequency (right).

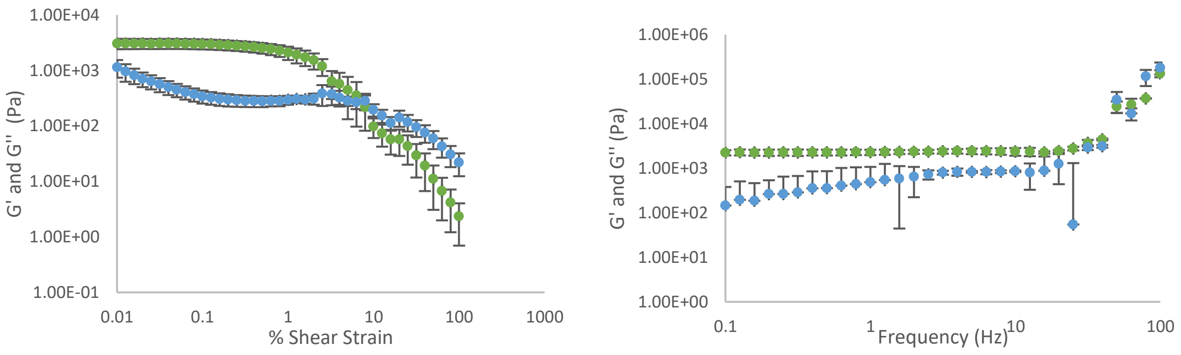

Figure S30. Elastic (G’, green line) and viscous (G’’, blue line) moduli of the 0.06 mmol $HCl_{(Square)}$ LMWG object, with increasing shear strain (left) and frequency (right).

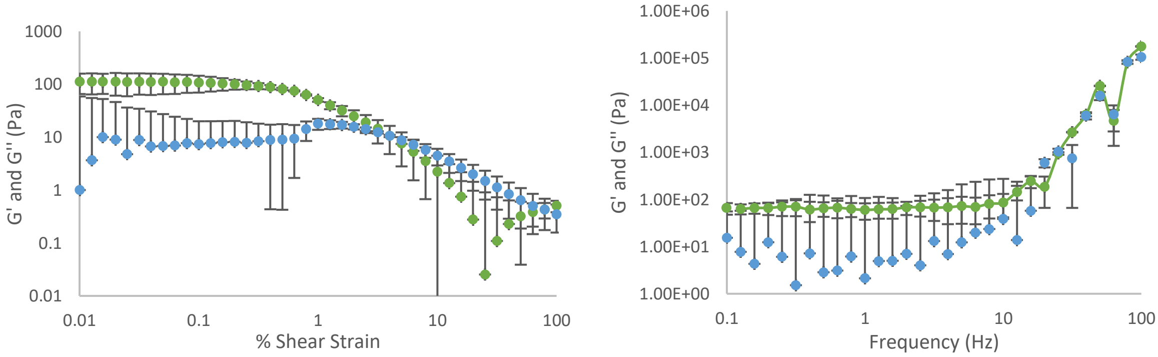

Figure S31. Elastic (G’, green line) and viscous (G’’, blue line) moduli of the 0.144 mmol $HCl_{(Square)}$ LMWG object, with increasing shear strain (left) and frequency (right).

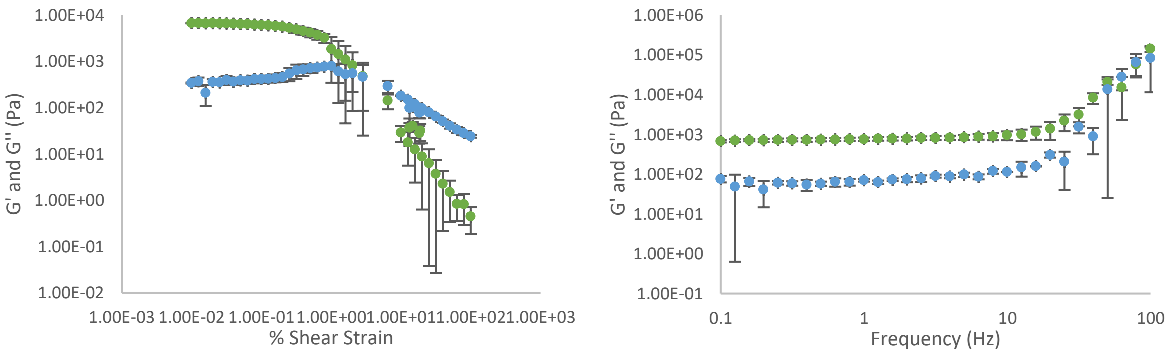

Figure S32. Elastic (G’, green line) and viscous (G’’, blue line) moduli of the 0.20 mmol $HCl_{(Square)}$ LMWG object, with increasing shear strain (left) and frequency (right).

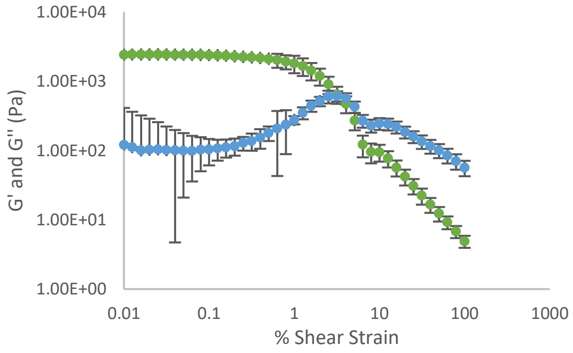

Figure S33. Elastic (G’, green line) and viscous (G’’, blue line) moduli of the 0.03 mmol $GdL_{(Square)}$ hybrid object, with increasing shear strain (left) and frequency (right).

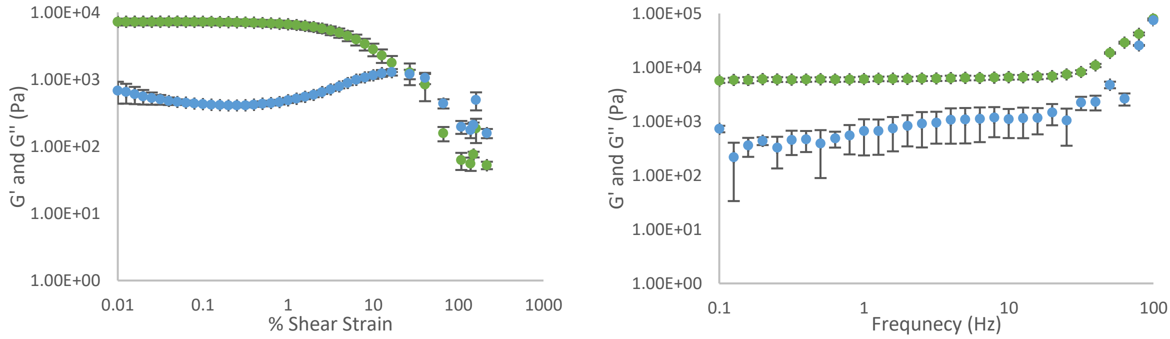

Figure S34. Elastic (G’, green line) and viscous (G’’, blue line) moduli of the 0.03 mmol $GdL_{(Square)}$ hybrid outer, with increasing shear strain (left) and frequency (right).

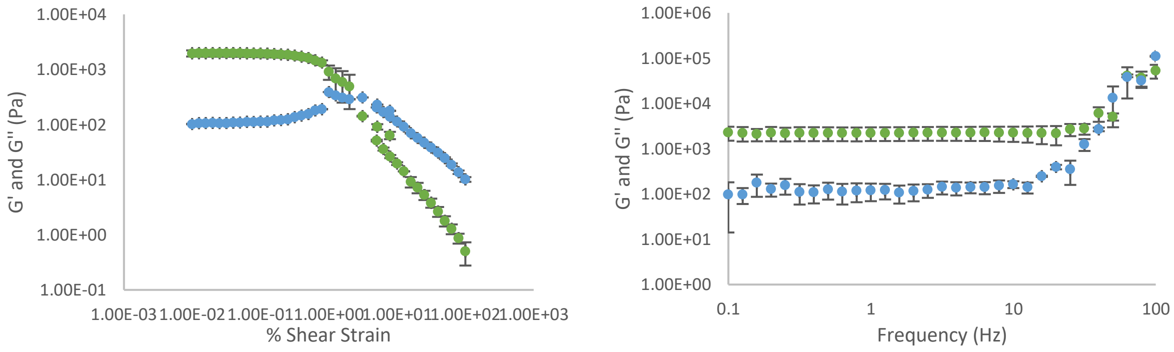

Figure S35. Elastic (G’, green line) and viscous (G’’, blue line) moduli of the 0.06 mmol $GdL_{(Square)}$ hybrid object, with increasing shear strain (left) and frequency (right).

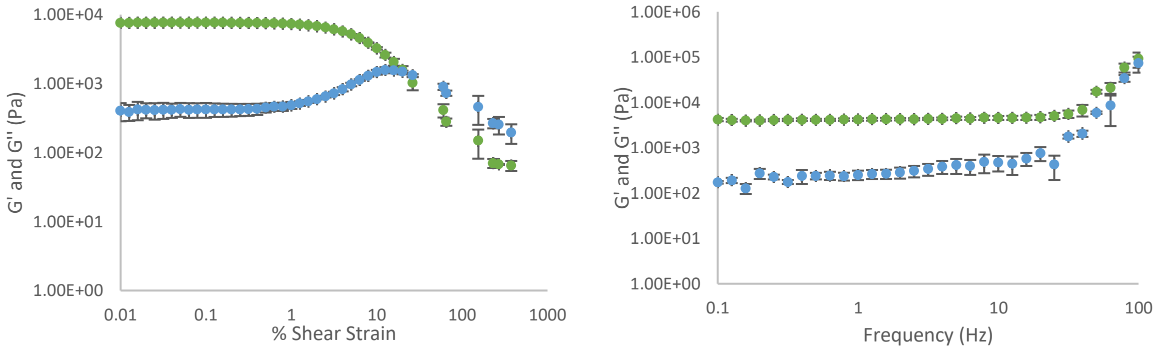

Figure S36. Elastic (G’, green line) and viscous (G’’, blue line) moduli of the 0.06 mmol GdL$_{(Square)}$ hybrid outer, with increasing shear strain (left) and frequency (right).

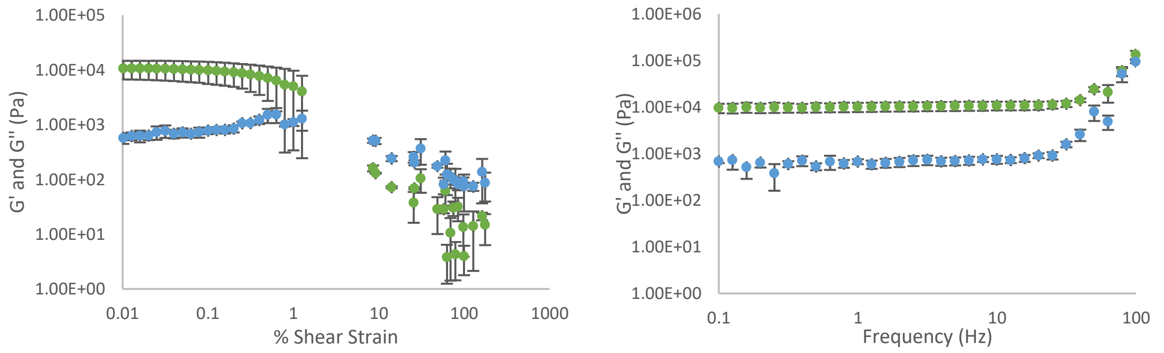

Figure S37. Elastic (G’, green line) and viscous (G’’, blue line) moduli of the 0.144 mmol GdL$_{(Square)}$ hybrid object, with increasing shear strain (left) and frequency (right).

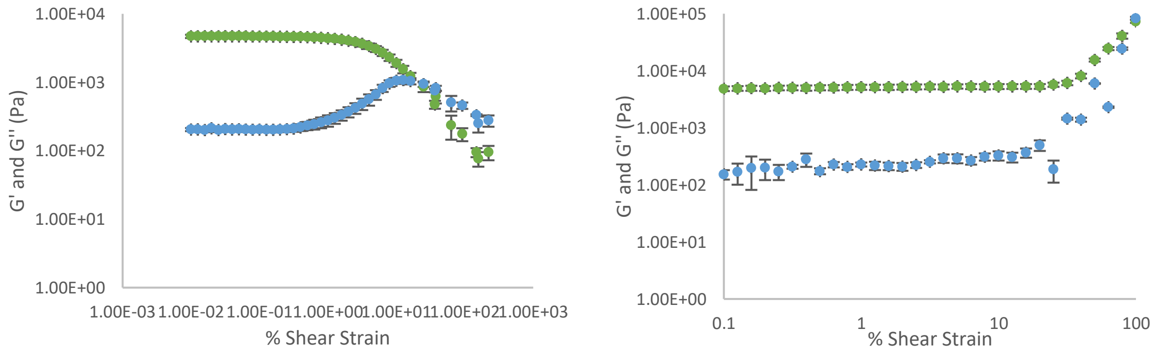

Figure S38. Elastic (G’, green line) and viscous (G’’, blue line) moduli of the 0.144 mmol GdL$_{(Square)}$ hybrid outer, with increasing shear strain (left) and frequency (right).

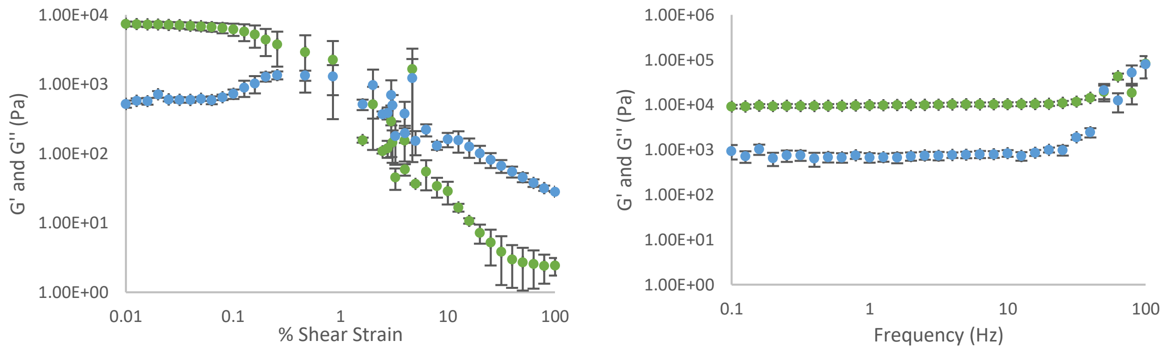

Figure S39. Elastic (G’, green line) and viscous (G’’, blue line) moduli of the 0.20 mmol GdL$_{(Square)}$ hybrid object, with increasing shear strain (left) and frequency (right).

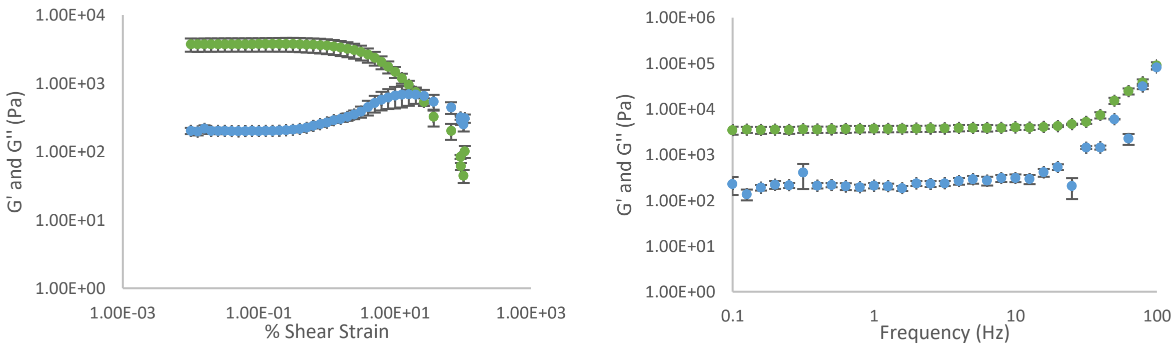

Figure S40. Elastic (G’, green line) and viscous (G’’, blue line) moduli of the 0.20 mmol GdL$_{(Square)}$ hybrid outer, with increasing shear strain (left) and frequency (right).

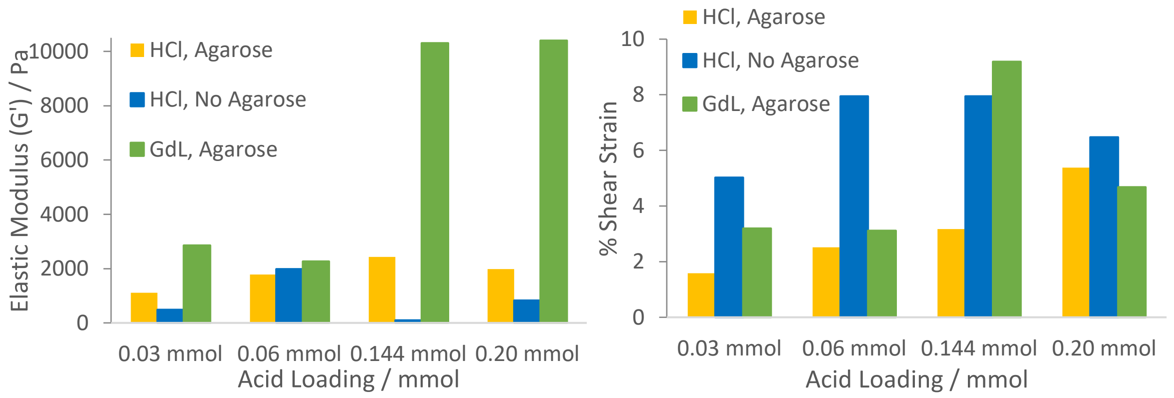

Figure S41. The elastic (G’) modulus (left) and % shear strain of the HCl$_{(Square)}$ hybrid (yellow), HCl$_{(Square)}$ LMWG (blue) and GdL$_{(Square)}$ hybrid (green) objects.

S8. References

1. B. O. Okesola and D. K. Smith, *Chem. Commun.* 2013**,** **49**, 11164-11166.
2. D. J. Cornwell, B. O. Okesola and D. K. Smith, *Soft Matter* 2013**,** **9**, 8730-8736.
